# Role of a Contactin multi‐molecular complex secreted by oligodendrocytes in nodal protein clustering in the CNS

**DOI:** 10.1002/glia.23681

**Published:** 2019-07-22

**Authors:** Anne‐Laure Dubessy, Elisa Mazuir, Quentin Rappeneau, Sokounthie Ou, Charly Abi Ghanem, Kevin Piquand, Marie‐Stéphane Aigrot, Melina Thétiot, Anne Desmazières, Eric Chan, Matt Fitzgibbon, Mark Fleming, Raul Krauss, Bernard Zalc, Barbara Ranscht, Catherine Lubetzki, Nathalie Sol‐Foulon

**Affiliations:** ^1^ Sorbonne Université, Inserm, CNRS, UMR7225 Institut du Cerveau et de la Moelle épinière, ICM Paris France; ^2^ Assistance Publique‐Hôpitaux de Paris GH Pitié‐Salpêtrière Paris France; ^3^ Vertex Pharmaceuticals Incorporated Boston Massachusetts; ^4^ Disarm Therapeutics Cambridge Massachusetts; ^5^ Sanford Burnham Prebys Medical Discovery Institute La Jolla California

**Keywords:** Contactin‐1, Na_v_ channels, nodal clusters, oligodendrocytes, Phosphacan, Tenascin‐R

## Abstract

The fast and reliable propagation of action potentials along myelinated fibers relies on the clustering of voltage‐gated sodium channels at nodes of Ranvier. Axo‐glial communication is required for assembly of nodal proteins in the central nervous system, yet the underlying mechanisms remain poorly understood. Oligodendrocytes are known to support node of Ranvier assembly through paranodal junction formation. In addition, the formation of early nodal protein clusters (or prenodes) along axons prior to myelination has been reported, and can be induced by oligodendrocyte conditioned medium (OCM). Our recent work on cultured hippocampal neurons showed that OCM‐induced prenodes are associated with an increased conduction velocity (Freeman et al., 2015). We here unravel the nature of the oligodendroglial secreted factors. Mass spectrometry analysis of OCM identified several candidate proteins (i.e., Contactin‐1, ChL1, NrCAM, Noelin2, RPTP/Phosphacan, and Tenascin‐R). We show that Contactin‐1 combined with RPTP/Phosphacan or Tenascin‐R induces clusters of nodal proteins along hippocampal GABAergic axons. Furthermore, Contactin‐1‐immunodepleted OCM or OCM from *Cntn1*‐null mice display significantly reduced clustering activity, that is restored by addition of soluble Contactin‐1. Altogether, our results identify Contactin‐1 secreted by oligodendrocytes as a novel factor that may influence early steps of nodal sodium channel cluster formation along specific axon populations.

## Main Points

- Oligodendrocyte secreted factors induce prenode formation on hippocampal GABAergic axons.
- Proteomic analysis of OCM to select clustering candidates.
- Contactin‐1 in association with Tenascin‐R or Phosphacan is responsible for prenodal clustering.

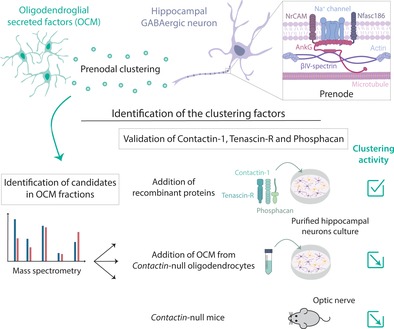

## 1 INTRODUCTION

Clustering of voltage‐gated sodium channels (Na_v_) at nodes of Ranvier is crucial for saltatory transmission and regeneration of action potentials along myelinated fibers. At nodes in the central nervous system, Na_v_ channels which consist of a pore‐forming α subunit associated with two accessory ß subunits, are associated with cell adhesion molecules (CAM) (i.e., Nfasc186 and NrCAM), a cytoskeletal scaffold (i.e., AnkyrinG and ßIV spectrin), and extracellular matrix (ECM) proteins (Salzer, 2003). Assembly of the nodes of Ranvier depends on neuro‐glial interactions which differ between the peripheral nervous system (PNS) and the central nervous system (CNS) (Freeman, Desmazières, Fricker, Lubetzki, & Sol‐Foulon, 2016; Rasband & Peles, 2015; Sherman & Brophy, 2005). In the PNS, Gliomedin and NrCAM expressed by Schwann cells at the edges of forming myelin segments initiate nodal protein clustering through contacts with axons forming heminodes that then fuse through paranodal junction‐dependent mechanisms to form mature nodes (Ching, Zanazzi, Levinson, & Salzer, 1999; Feinberg et al., 2010; Vabnick, Novaković, Levinson, Schachner, & Shrager, 1996). In the CNS, multiple complementary molecular mechanisms are proposed to trigger Na_v_ clustering (Susuki et al., 2013). First, the diffusion barrier established through the cytoskeletal assembly at paranodes has a prominent role in node formation (Amor et al., 2017; Brivio, Faivre‐Sarrailh, Peles, Sherman, & Brophy, 2017; Rasband et al., 1999; Susuki et al., 2013; Zonta et al., 2008). Paranodal associations can promote Na_v_ clustering in the absence of Nfasc186 as demonstrated by genetic inactivation of *Nfasc* gene expression (Zonta et al., 2008). Second, ECM protein interactions with Nfasc186 are sufficient to promote Na_v_ clustering independent of paranodal junction formation (Bhat et al., 2001; Boyle et al., 2001; Dupree, Girault, & Popko, 1999; Susuki et al., 2013; Zonta et al., 2008). Third, both Na_v_ α and ß subunits, as well as Nfasc186, bind to AnkyrinG, which is crucial for their clustering at the node (Gasser et al., 2012; Jenkins et al., 2015; Susuki et al., 2013). In addition to this tri‐partite mechanism, soluble oligodendrocyte‐derived factors present in oligodendrocyte conditioned medium (OCM) are able to induce nodal protein clustering in neuronal cultures, in the absence of oligodendroglial cell contact and paranodal junctions. This was first demonstrated by the Barres group on retinal ganglion cells (Kaplan et al., 1997; Kaplan et al., 2001) and more recently reproduced by our group on hippocampal GABAergic neurons (Freeman et al., 2015). Axons with such early nodal protein clusters, also referred to as prenodes, show increased conduction velocity as demonstrated by single cell electrophysiology in hippocampal cultures (Freeman et al., 2015). To date, however, the molecular identity of this oligodendroglial clustering activity has remained elusive.

To identify the oligodendroglial factor(s) inducing Na_v_ clustering, we conducted a proteomic analysis of OCM, and validated the activity of selected candidate proteins in purified hippocampal neuron cultures. Here, we report that Contactin‐1 (also referred to as CNTN), secreted by oligodendrocytes has a central role in OCM‐induced clustering activity. Our data provide evidence that a multi‐molecular complex consisting of a combination of recombinant CNTN with ECM proteins RPTP/Phosphacan or Tenascin‐R is sufficient to induce nodal protein clusters on hippocampal GABAergic neurons. Depletion of CNTN from OCM using either immunoprecipitation or glycosyl‐phosphatidylinositol (GPI) cleavage inhibition, significantly reduces the clustering activity. Furthermore, we show that OCM from oligodendrocytes with inactivated *Cntn1* gene expression displayed reduced clustering activity, and that this deficiency is rescued by addition of soluble CNTN. Finally, neither Nfasc186 nor NrCAM, are required for OCM induced‐clustering, although the presence of elongated clusters suggests a possible role for NrCAM in the stabilization of the complex.

## 2 MATERIALS AND METHODS

### 2.1 Animals

The care and use of rats and mice in all experiments conformed to institutional policies and guidelines (UPMC, INSERM, and European Community Council Directive 86/609/EEC). The following rat and mouse strains were used in this study: Sprague–Dawley or Wistar rats (Janvier Breeding Center), PLP‐GFP mice (Spassky et al., 2001) and *NrCAM* ^−^/^−^ (B6.129P2‐*NrCAM* ^*tm1Fgt/orl*^) mice (EMMA center, CNRS, TAAM). The derivation of mice homozygous for the mutant contactin‐1 (*Cntn1*) allele was described previously (Berglund et al., 1999).

### 2.2 Cell cultures

#### 2.2.1 Culture media

NM, neurobasal medium (21103–049; Gibco) supplemented with 0.5 mM L‐glutamine, B27 (1×; Invitrogen), and penicillin–streptomycin (100 IU/mL each). Bottenstein‐Sato medium (BS): DMEM Glutamax supplemented with transferrin (50 μg/mL), albumin (50 μg/mL), insulin (5 μg/mL), progesterone (20 nM), putrescine (16 μg/mL), sodium selenite (5 ng/mL), T3 (40 ng/mL), T4 (40 ng/mL), PDGF (10 ng/mL).

##### *Neuronal cultures*

Mixed hippocampal cultures (containing neurons and glial cells) and purified hippocampal neuron cultures were obtained from E18 animals as previously described and characterized (Freeman et al., 2015). To obtain purified neuron culture, anti‐mitotic agents (FdU and U 5 μM) were added 24 hr after dissection, and then removed after 36 hr. OCM (500 μL/well) was added to purified hippocampal neurons at 3 days in vitro (DIV) or at later time points as indicated. One‐third of the medium was changed with neurobasal medium (NM) at 7 DIV, and then twice a week. Unless otherwise specified, cells were fixed at 17 DIV.

##### *Glial cell cultures and OCM*

Glial cell cultures were prepared form cerebral cortices from P2 Wistar rats as previously described (Freeman et al., 2015). Cortices were dissected free of meninges, incubated 35 min in papain (30 U/mL; Worthington), supplemented with L‐cystein (0.24 mg/mL) and DNase (50 μg/mL) in DMEM at 37° and then mechanically homogenized and passed through a 0.70 μm filter. Cells were re‐suspended in DMEM with 10% FCS and 1% penicillin–streptomycin. After 7 to 14 DIV, flasks were shaken overnight at 230 rpm at 37°C. Collected cells were then incubated in dishes for 15 min and non‐adherent cells were then collected and centrifuged in DMEM for 5 min at 1500 rpm and re‐suspended and seeded on Polyethyleneimine (PEI)‐coated dishes at a density of 1.5 × 10^5^/cm^2^ in BS medium with 0.5% PDGF. Media was replaced with NM and collected after 48 hr. OCM was filtered through a 0.22 μm filter. Protein concentration (4.1 μg/μl ± 2.4, mean ± *SD* of four OCMs) was measured using the BCA protein assay (Pierce). Brains from P0 to P2 *CNTN* ^*+/+*^ or *CNTN* ^*−/−*^ pups were harvested in the laboratory of Dr. B. Ranscht, La Jolla, and sent at 4°C in Hibernate™ medium (Gibco) and treated as rat cerebral cortices. After 7 to 10 DIV, depending on density, medium was replaced by BS medium. After 48 hr, the first conditioned medium was collected, and culture medium was renewed, allowing the second collection after 48 hr.

Oligodendrocytes isolated at the later stage of development were obtained by FACS‐sorting of dissociated brains from *PLP‐GFP* mice at P9 (instead of P2), and were cultured with neurobasal medium for 48 hr to produce mature OCM.

#### 2.2.2 Tissue sections

Optic nerves from mice were dissected in 0.1 M phosphate buffer (PB) immediately after mice were killed. Nerves were fixed in 2% PFA (diluted in PBS 1X; pH 7.2), for 30 min, cryoprotected in 30% sucrose over‐night, frozen in OCT (Tissue‐Tek) and then cryosectioned (LeicaCM3050) at 6 μm.

#### 2.2.3 Antibodies and immunofluorescence

Cell cultures were fixed with 4% PFA for 10 min, or, for Na_v_ channel staining, with 1% PFA, for 10 min at RT and then incubated with methanol for 10 min at −20°C. Coverslips were then washed with PBS 1X. After fixation, cells were incubated with blocking buffer (PBS 1X, normal goat serum [NGS] 5%, Triton 0.1%) for 15 min and then with primary Ab diluted in blocking buffer for 2 hr at RT or at 4°C overnight. Coverslips were then washed and incubated with secondary Ab at RT for 1 hr. After last wash, coverslips were mounted with Fluoromount‐G with DAPI (Southern Biotech). Optic nerve sections were incubated with blocking buffer (PB 1X, NGS 10%, Triton 0.3%) for 1 hr and then with primary Ab overnight at 4°C. Slides were then washed and incubated with secondary Ab at RT for 1 hr, washed again and mounted with Fluoromount‐G with DAPI.

The following primary Abs were used for immunolabeling. Mouse mAbs included anti‐AnkG (clone N106/36; 1:50) and Nfasc (pan, external; cloneA12/18; 1:100) from Antibodies Incorporated (Neuromab). Anti‐Nav (pan; clone K58/35; 1:300) was from Sigma. Anti‐GAD67 (clone 1G10.2; 1:400) was from Millipore. Anti‐TNR (clone 619, 1:150) was from R&D system. Anti‐O4 (IgM; 1:5 hybridoma) was also used. Rabbit polyclonal Abs included anti‐NG2 (1:500; Chemicon), neurofilament M, C‐terminus (1:400; Millipore), Caspr (1:400; Abcam), Nfasc (pan; 1:100; Abcam), NrCAM (1:1500, Abcam), AnkG (1:500; provided by François Couraud INSERM, UMRS952, Paris). We also used chicken anti‐ GFP (1:400; Millipore), chicken anti‐MBP (1:500; Millipore) and rat anti‐PLP (1:10; hybridoma). Secondary Abs were Alexa Fluor 488, 594, or 647 goat anti‐rabbit or anti‐mouse IgG2a, IgG1, or IgM, anti‐chicken, anti‐rat from Invitrogen (1:1000), and Alexa488‐ conjugated anti‐human Fc antibody from Jackson Immunoresearch laboratories (1:1000).

The following Abs were used for Western Blotting (WB): Sheep anti‐CNTN (1:1000) from R&D systems, mouse anti‐Actin (1:4000) and anti‐GAPDH (clone 6C5; 1:4000) from Millipore, mouse anti‐TNR (clone 619, 1:4000) from R&D system. Secondary antibodies included rabbit anti‐sheep HRP (1:3000) from Life technologies, goat anti‐mouse HRP (1:4000) Sigma‐Aldrich or Invitrogen.

#### 2.2.4 Recombinant proteins

Unique or combination of recombinant proteins were added in culture media on hippocampal neurons at 3 to 14 DIV. Recombinant proteins were reconstituted at 100 μg/mL in sterile PBS, and unless otherwise specified, their final concentration in culture was 1.4 μg/mL. The following recombinant proteins from R&D systems were used: human contactin‐Fc, human NrCAM‐Fc and mouse NrCAM, human ChL‐1, human olfactomedin‐2 and human Tenascin‐R.

Plasmid containing the carbonic anhydrase domain (CAH) plus fibronectin domains of RPTPß fused to the Fc of human IgG1 was a gift from O. Peles and purified rRPTPß‐Fc was a gift from J. Devaux.

The following recombinant growth factors from Peprotech were used: BDNF and GDNF (20 ng/mL) and IGF‐1 (100 ng/mL).

Contactin‐Fc (0.7 μg/mL) was pre‐clustered by incubation with antihuman Fc (1 μg/mL, Jackson Immunoresearch laboratories) in Neurobasal Medium for 30 min at 37°C; pre‐clustered CNTN was added for 2 hr to purified neurons after 14 DIV and then incubated with Neurobasal culture medium until fixation at 17 DIV.

#### 2.2.5 Drugs

When indicated, the following drugs were incubated with oligodendrocyte cultures for 1 hr: edelfosine, a synthetic phospholipid analog that inhibits phosphatidylinositol phospholipase C (Tocris, CAS number: 77286–66‐9, stock 5 mM in water, 10 μM final); U73122, a phospholipase C inhibitor (Sigma, CAS number: 112648–68‐7, stock 5 mM in DMSO, 5 μM final). Then, medium was replaced with neurobasal medium for 48 hr to produce OCM.

#### 2.2.6 Design and transfection of miRNA

To generate Nfasc miRNA constructs, we used the Block‐it PolII miR RNAi Expression Vector kit (K4936‐00). Two different miRNA sequences were designed to knock down the expression of Nfasc: TGC TGT AAA GGA TCA CCC TGA TGA G CG TTT TGG CCA CTG ACT GAC GCT CAT CAG TGA TCC TTTA and CCT GTA AAG GAT CAC TGA TGA GCG TCA GTC AGT GGC CAA AAC GCT CAT CAG GGT GAT CCT TTAC. They were then cloned into the supplied vector (pcDNA 6.2‐GW/EmGFP‐miR). A plasmid expressing miRNA predicted not to target any known vertebrate gene was used as a control. A GFP marker was co‐expressed to identify transfected cells. Culture medium of mixed hippocampal cultures at 6 DIV was replaced with PS‐free medium 2 hr before transfection. Nfasc186 miRNA (0.4 μg DNA/well) were transfected into the cells using lipofectamine 2000 transfection reagent following manufacturer instructions (Invitrogen, ThermoFisher Scientific, Waltham, Massachussets).

#### 2.2.7 Image acquisition and quantitative analysis

Images were acquired using an Olympus FV‐1200 Upright Confocal Microscope or a Zeiss Axio‐Imager‐Apotome. Z‐series of optical sections were performed at 0.3 μm increment, and blue, green, red, and far‐red fluorescence were acquired sequentially. Maximum orthogonal projection of images and plot profiles of immunofluorescence intensity were carried out using Fiji software (NIH, Bethesda, Maryland). At least 50 GABAergic neurons per coverslip, identified by GAD67^+^ immunostaining, were counted, and the percentage of GAD67^+^ neurons with Na_v_/AnkG clusters was determined for at least two coverslips per condition. Results were expressed as mean ± *SEM* of at least three independent experiments. *p*‐value between the tested condition and control condition were calculated using the Mann–Whitney test, unless otherwise specified. A Na_v_ cluster was defined by its length and mean intensity of area (i.e., size of clusters varied from 1 to 8 μm, and mean value of cluster area was at least 2.5 that of the adjacent part of the axon). Cluster length was measured on Na_v_ plot profiles, and was measured at half‐height of the fluorescence peak. For the quantification of cluster lengths in KO‐OCM and WT‐OCM treated neurons, >600 clusters in three different experiments were quantified per condition, *p*‐value was hence calculated using a Student *t*‐test.

Na_v_ clusters density has been quantified on images acquired from optic nerve sections immunostainings as the number of clusters with a fluorescence intensity at least three times higher than the background level and an area comprised between 0.5 and 4 μm^2^ over the tissue area.

#### 2.2.8 Immunoprecipitation

Immunoprecipitation of CNTN from OCM was performed using IgG4 anti‐CNTN or IgG4 CTRL human antibodies (kind gift from J. Devaux) (1:100). OCM samples were incubated with antibodies overnight at 4°C, then Dynabeads^©^ Protein G (Novex^©^) were added to the medium and incubated 1.5 hr at 4°C. Using a magnet (DynaMag™), Dynabeads‐Ab‐CNTN/CTRL complexes were then separated from OCM. CNTN/CTRL depleted‐OCM was either added on neurons or submitted to a second cycle of incubation with anti‐CNTN/CTRL antibody to improve the depletion. Dynabeads‐Ab‐CNTN/CTRL complexes were then eluted and analyzed by WB.

#### 2.2.9 Western blot analysis

Protein extracts were obtained from lysates of cell cultures, OCM, or eluted from Dyneabeads following immunoprecipitation. They were separated on Bis‐Tris10% gels (ThermoSci) and transferred onto nitrocellulose membranes. Membranes were incubated with 1X PBST with 5% nonfat dry milk for 1 hr RT, then incubated overnight at 4°C with a primary antibodies solution in PBS‐Milk. Proteins of interest were detected with HRP‐conjugated secondary antibodies and visualized with the Pierce ECL Western blotting substrate (Thermo Scientific, Rockford, IL), according to the provided protocol.

#### 2.2.10 OCM fractionation and proteomic analyses

About 45–50 mL of OCM or control media was concentrated using a 10 K MWCO Vivaspin‐20 to a final volume of 1.5 mL. Each sample was diluted to 3.5 mL with Buffer‐A (25 mM Tris pH 7.6) to reduce the salt concentration and loaded on a Mono‐Q (0.5 × 5 cm) anion exchange column (GE Life Sciences) equilibrated in Buffer‐A at 1 mL/min. The column was washed to baseline with Buffer‐A and proteins were eluted with a NaCl gradient as follows; 0–0.25 M NaCl over 30 min, 0.25–0.5 M NaCl in 5 min. Any remaining proteins were stripped off the column with 2 M NaCl. One‐milliliter fractions were collected and assayed for nodal formation activity, reserving 100 μL for subsequent protein identification. Peak activity fractions and corresponding fractions from control media were reduced by addition of DTT to 10 mM and incubated at 55°C for 30 min. Samples were alkylated by addition of Iodoacetamide to 30 mM and incubated at room temperature in the dark for 30 min. Samples were digested with trypsin overnight at 37°C. Digestion was quenched by addition of trifluoroacetic acid (TFA) to 0.5% and loaded on a C18 ultramicro spin column (The nest group). The spin columns were washed with 0.2% TFA and eluted with 0.2% TFA + 25% acetonitrile, followed by 0.2% TFA + 60% acetonitrile. The eluates were combined and dried in a speed‐vac centrifuge. LC/MS/MS was performed on an Orbitrap Elite (Thermo Fisher Scientific, Bremen, Germany) with a Nanoflex source (Thermo Fisher Scientific), coupled to a Dionex Ultimate 3,000 UHPLC system (Thermo Fisher Scientific). Each sample was injected onto a PepMap Acclaim trap column (0.075 × 20 mm^2^, 5 μm C18 particles; Thermo Fisher Scientific) at 5% acetonitrile and 0.1% formic acid loaded at 30 μL/min for 8 min; peptides were then separated on a PepMap Acclaim analytical column (0.075 × 500 mm^2^, 3 μm C18 particles; Thermo Fisher Scientific) at 300 nL/min on a linear gradient of 5–50% acetonitrile in 0.1% formic acid over 100 min. The 10 most intense precursor ions (m/z 300–2000 and charge state or 2+ or 3+) were isolated at a width of 1.5 Da, and selected for MS/MS using collision‐induced dissociation with a normalized collision energy of 38%. Dynamic exclusion option was enabled with a repeat count of one and exclusion duration of 30 s.

Raw data were proceeded using Proteome Discoverer v1.4 (Thermo Fisher Scientific) with Mascot v2.4 (Matrix Science, London, UK) against Uniprot (UP Human without isoform, 14.3 release version), following LC–MS/MS analysis. Protein identification was accepted with a 1% protein FDR threshold as calculated by the Percolator node; a minimum of two peptides meeting the criteria were required for protein identification.

### 2.3 Statistical analyses

*p*‐values for the comparison of the effect of r‐proteins, depletion experiments, KO versus WT experiments, were calculated using Student *t*‐test when number of n was >30 (comparison of cluster length) or Mann–Whitney test when *n* was <30. For PLC inhibitors analysis, Dunn's multiple comparison test was used to compare PLC inhibitors to control condition.

## 3 RESULTS

### 3.1 Na_v_ clustering activity of oligodendroglial‐secreted factors on GABAergic hippocampal neurons

**Figure 1:**
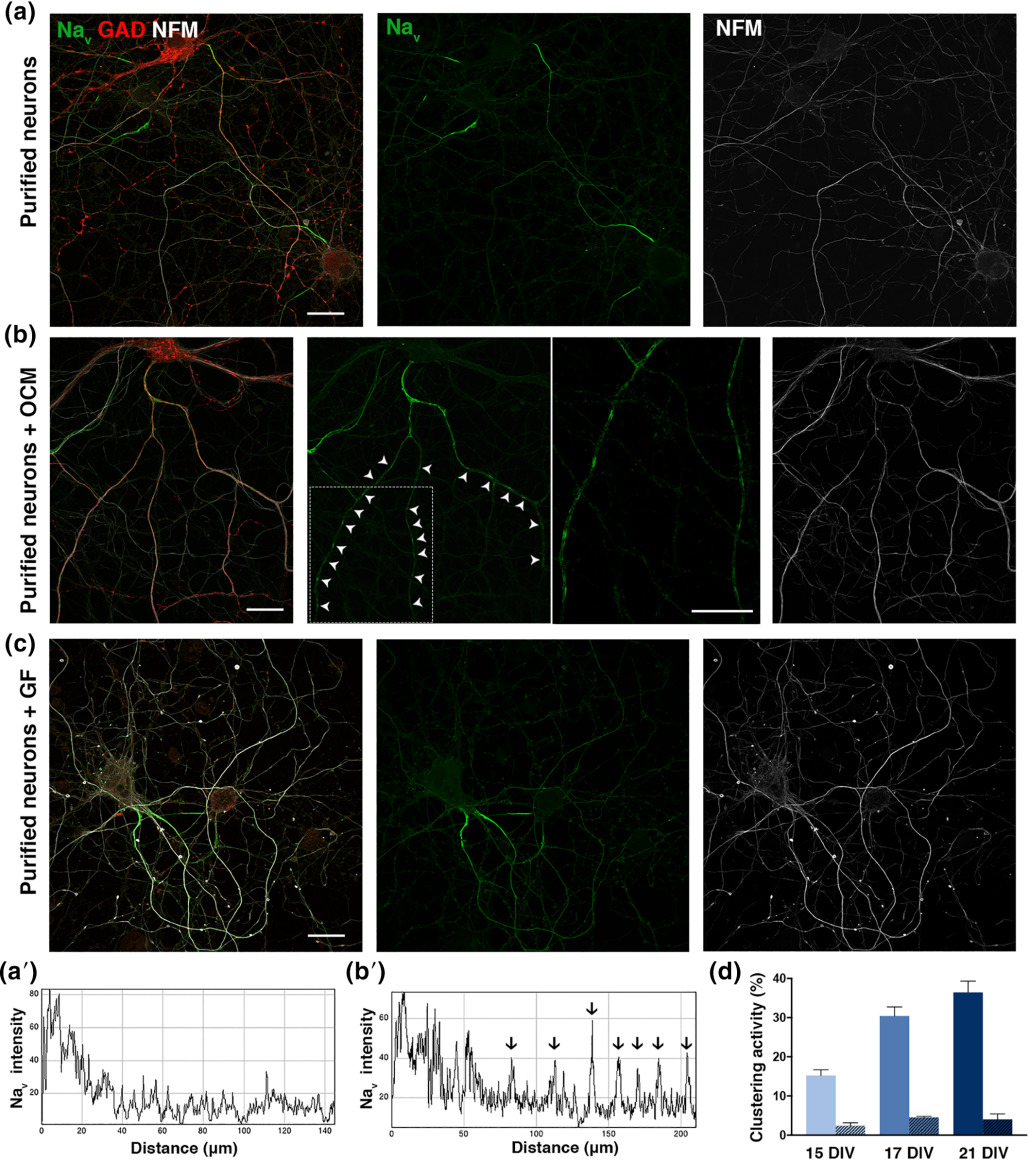
OCM promotes Na_v_ clustering on GABAergic hippocampal neurons in culture. (a, b, c) immunostainings of purified hippocampal neurons cultured in the absence of OCM (a), presence of OCM (b) or growth factors (GF); that is, IGF‐1, BDNF, and GDNF (c) fixed at 17 DIV. The axon initial segment (AIS) is detected in all conditions (a–c), but clusters of Na_v_ (green) are only detected in the presence of OCM (b) on GABAergic axons (GAD67^+^; red). The periodic clusters of Na_v_ channels are shown at a higher magnification of the framed part. Neurites are stained with an antibody targeting neurofilament M (NFM; white). Scale bars 25 μm. (a′, b′) Fluorescence intensity profiles correspond to axonal Na_v_ immunolabeling from (a and b); individual peaks in b′ (arrows) represent Na_v_ clusters. (d) Clustering activity represents the percentage of GABAergic axons (GAD67^+^, AIS^+^) having at least two Na_v_ clusters and is quantified after 15, 17, or 21 DIV. Purified neurons were cultured in the absence of OCM (non‐hatched box), or with OCM (hatched box) added at 3 DIV. The mean ± *SEM* of three independent experiments is shown. For each experiment, at least 100 neurons were analyzed. OCM, oligodendrocyte conditioned medium; DIV, days in vitro

Purified hippocampal neurons, in cultures which are virtually devoid of oligodendrocytes and contain less than 5% of astrocytes, form only very few nodal clusters in vitro, whereas addition of OCM to purified hippocampal neurons after 3 DIV induces along the axon nodal protein clusters consisting of Na_v_ channels associated with Nfasc186 and AnkyrinG (Freeman et al., 2015). A cluster is defined by a Na_v_ immunolabeling intensity ≥2.5‐fold over its basal level along the axon (identified by the presence of an axon initial segment [AIS] in Figure 1a,b) and a length comprised between 1 and 8 μm (arrowheads and arrows in Figure 1b,b′). As previously reported, these clusters are restricted to GABAergic neurons (Freeman et al., 2015). Only few axons with Na_v_ clusters were detected at 15 DIV, while the percentage of axons with clusters significantly increased by 17 and 21 DIV (mean ± *SEM*: 15.2 ± 1.5%, 30.4 ± 2.3%, and 36.4 ± 2.9%, respectively) (Figure 1d). This late response of hippocampal neurons to OCM addition suggests that neuronal maturation is necessary for the Na_v_ clustering. Increased neuronal expression of the intermediate filament protein neurofilament M (NFM) upon OCM treatment was observed suggesting an effect of OCM on GABAergic neuron maturation (Figure 1a,b). In addition, we measured axonal lengths at 6 DIV in control purified neurons and neurons treated with either OCM or recombinant growth factors (GDNF, BDNF, and IGF1). As shown in Figure S1, both treatment conditions induced a significant increase in axonal lengths of both hippocampal GABAergic and glutamatergic pyramidal neurons. However, the possibility that OCM‐induced nodal protein clustering is solely related to OCM‐induced neuronal maturation was ruled out by the fact that addition of GDNF, BDNF, and IGF1 to purified neurons did not promote nodal protein clustering at 17 DIV (mean ± *SEM*, 2.7 ± 0.3%) (Figure 1c).

In these experiments, OCM was produced from cells of the oligodendrocyte lineage mostly expressing NG2 and O4 markers, hence corresponding to immature oligodendrocytes (Figure S2a). To address whether the state of oligodendroglial maturation influences OCM‐clustering activity (quantified as the percentage of axons with Na_v_ clusters among GABAergic neurons), we produced OCM from either less differentiated oligodendroglial cells (increased percentage of NG2 expressing cells) or from oligodendrocytes isolated at a later stage of development (purified by flow cytometry from PLP [proteolipid protein]‐GFP mouse brains) (Spassky et al., 2001) and added these different OCMs to hippocampal neurons. No significant differences in clustering activity were observed (Figure S2b,c). This establishes that the soluble oligodendroglial factor(s) responsible for Na_v_ clustering can be produced by cells throughout oligodendroglial lineage progression.

### 3.2 Identification of oligodendroglial factors promoting Na_v_ clustering activity

**Figure 2:**
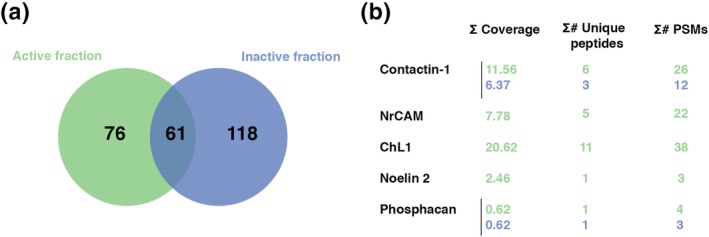
Proteomic analysis of OCM fractions. (a) Representative diagram of partially overlapping proteomic content of an active (13, green) and an inactive (15, blue) fraction, with 61 proteins present in both fractions, 76 proteins only in the active fraction and 118 only in the inactive fraction. (b) Short list of candidates retained for further testing. Indicated parameters are the percent coverage of the total protein sequence by the identified peptides (∑ coverage), the number of identified peptides that are unique to this protein (no overlap with other proteins in the database) (∑# unique peptides) and the peptide spectral match (∑# PSMs), that is, indicating how many times the mass spectra match that of the peptide sequence. Values corresponding to the active fraction (13) are in green and to the inactive fraction (15) are in blue. OCM, oligodendrocyte conditioned medium; DIV, days in vitro

To gain insight into the nature of OCM‐derived factors promoting Na_v_ clustering, we first established that OCM activity was abolished by heating at 70°C or by trypsin treatment, indicating that the clustering activity is proteinaceous. Centrifugation of OCM on filters with different MW cut‐offs indicated that 100, 90, and 60% of the activity was retained by a 30, 50, and 100 kDa filter, respectively. In addition, OCM activity was fully retained on an anion exchange column. These characteristics established that clustering activity is supported by protein(s) with a MW ≥50 kDa and bearing a global negative charge. We then eluted fractions of OCM from the anion exchange column and tested each fraction on purified hippocampal neurons for Na_v_ clustering activity. As control, activity of fractions from a culture medium not incubated with cells (control medium) was tested (Figure S3). From approximately 30 fractions in each experiment (*n* = 3), a peak of activity was reproducibly detected in 3–4 OCM elution fractions (Figure S3). Mass spectrometry analyses were performed on these fractions (named A 11 to A 13 in this representative experiment), either by pooling the fractions or by testing each one separately. Two OCM fractions without activity (A14–A15, in this representative experiment), as well as fractions of control medium with similar elution profile were also analyzed. As shown in Figure 2a, a total of 137 proteins were identified in the most active OCM fraction (A13, green) (Figure S4a). Amongst these, 61 proteins were also found in the OCM inactive fraction (A15, blue), while 76 were specifically detected in the active fraction (Figure S4b). From this list, candidates were selected by the criteria of known nodal expression, reported effects on axons and respective abundance in active versus inactive fractions for the proteins detected in both fractions. This generated a short list (Figure 2b) comprising Contactin‐1 (or Contactin, CNTN), a CAM belonging to the super‐family of immunoglobulins (Ig), with Ig and fibronectin domains and a glycosyl‐phosphatidyl‐inositol (GPI) anchor, NrCAM, close homolog to L1 (ChL1), Noelin‐2 (also named Olfactomedin‐2) which is an olfactomedin domain‐containing protein like Gliomedin (Tomarev & Nakaya, 2009), and Phosphacan, a cleavage product of RPTPß. In parallel, SILAC studies were also performed, based on comparison of OCM versus astrocyte‐CM or versus neuron‐CM which have a poor or no Na_v_ clustering activity, respectively (not shown). Amongst proteins upregulated in OCM, we noted the extracellular matrix protein Tenascin‐R (TNR), which is a binding partner of Nfasc186 (Volkmer, Zacharias, Nörenberg, & Rathjen, 1998) and Contactin‐1 (Nörenberg, Hubert, Brümmendorf, Tárnok, & Rathjen, 1995; Zacharias, Nörenberg, & Rathjen, 1999).

### 3.3 A multimolecular complex containing Contactin is sufficient to induce nodal protein clustering on hippocampal neurons

**Figure 3:**
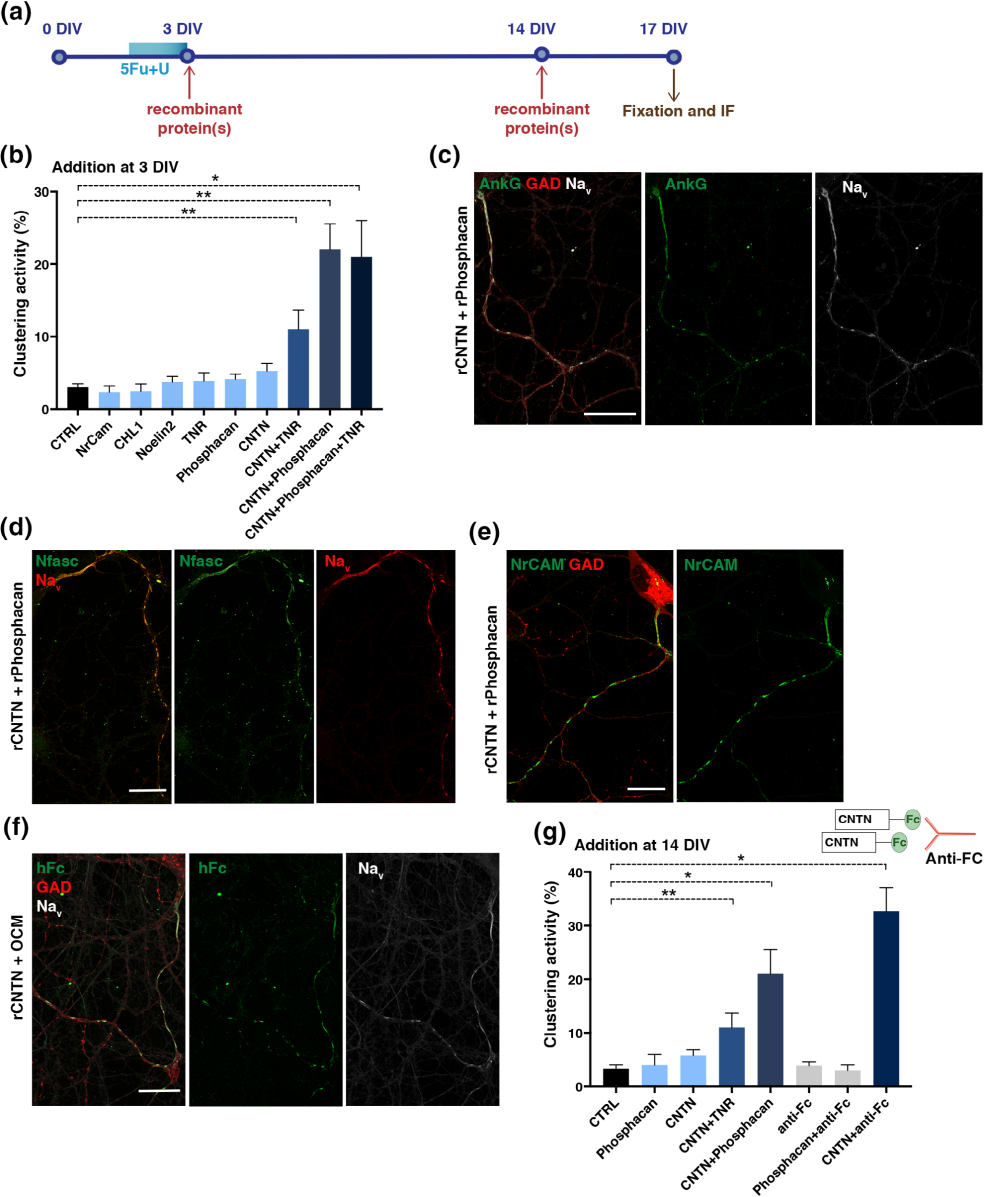
Contactin (CNTN) in combination with RPTP/Phosphacan or tenascin‐R (TNR) induces Na_v_ clustering on purified hippocampal neurons. (a) Schematic representation of experiment timing: recombinant proteins (1.4 μg/mL) were added at 3 or 14 DIV (red arrow) to purified hippocampal neurons obtained by antimitotic treatment (U + FdU, blue rectangle) for 36 hr. Cultures were fixed at 17 DIV for immunostainings (brown arrow). (b) Addition of single recombinant proteins to neuron cultures at 3 DIV induces weak Na_v_ clustering activity (evaluated as the percentage of GAD67^+^ axons with Na_v_ clusters) compared to control (i.e., untreated neurons, CTRL), while addition of CNTN combined with other ligands (RPTP/Phosphacan, TNR, or both) induced significant Na_v_ clustering compared to control, (*p = 0.0238 for CNTN‐RPTP/Phosphacan‐TNR vs. CTRL; **p = 0.0087 for CNTN‐TNR vs. CTRL and **p = 0.0048 for CNTN‐RPTP/Phosphacan vs. CTRL; Mann–Whitney test). The mean ± *SEM* of four independent experiments is shown. For each experiment, at least 100 neurons were analyzed. (c–e) Immunostainings of neurons treated with CNTN and RPTP/Phosphacan at 3 DIV showing Na_v_ clusters (white) colocalized with AnkG (green) (c), Na_v_ clusters (red) colocalized with Nfasc (green) (d), and NrCAM clusters (e) along GABAergic axons (GAD67^+^; red) (c and e); scale bars 25 μm. (f) rCNTN is detected at Na_v_ clusters at 17 DIV. OCM was supplemented with rCNTN and added on neurons at 3 DIV. rCNTN is identified by anti‐human Fc staining (hFc, green), colocalizing with Na_v_ clusters (white) along GAD67^+^ axon (red), scale bar 25 μm. (g) Significant Na_v_ clustering activity can be induced by late addition (14 DIV) of CNTN with RPTP/Phosphacan (*p = 0.0238), CNTN with TNR (**p = 0.0048), or CNTN pre‐clustered by incubation with antihuman Fc antibody (*p = 0.0238); Mann–Whitney test. The mean ± *SEM* of four independent experiments is shown. For each experiment, at least 100 neurons were analyzed. OCM, oligodendrocyte conditioned medium; DIV, days in vitro

To validate clustering activity of the selected candidates, recombinant (r) proteins were added to purified hippocampal neuron cultures at 3 DIV (Figure 3a). As none of the added (r) proteins had a significant clustering effect on its own, we hypothesized that protein complexes might be needed to promote clustering. Thus, we tested the activity of all combinations including CNTN, NrCAM, Noelin‐2, ChL1, RPTP/Phosphacan and TNR. Among them, as shown in Figure 3b and Figure S5a, only addition of rCNTN combined with rRPTP/Phosphacan or rTNR or both induced significant Na_v_ clustering activity (i.e., mean ± *SEM*: 22 ± 4%, *p* = 0.005; 11 ± 3%, *p* = 0.009 and 21 ± 5%, *p* = 0.025, respectively). CNTN binds both to RPTP/Phosphacan and TNR (Peles et al., 1995; Rathjen, Wolff, & Chiquet‐Ehrismann, 1991), suggesting possible formation of a multimolecular complex with CNTN. Compared to the full activity of OCM, rCNTN combined with rRPTP/Phosphacan or rTNR induced 72 and 36% of OCM‐induced clustering, respectively. With these combinations, as with OCM, other molecules of the nodal complex (i.e., AnkyrinG, Nfasc186, and NrCAM) were also recruited and colocalized with Na_v_ clusters on GABAergic neurons (Figure 3c,d,e).

We next determined the localization of CNTN on purified neurons treated at 3 DIV with OCM and Fc‐tagged rCNTN. Staining with the anti‐Fc antibody showed rCNTN colocalized with Na_v_ clusters (Figure 3f) indicating that soluble CNTN is stabilized in a complex at prenodes. To address whether CNTN‐interacting proteins also associate with prenodes, purified neurons treated with OCM were incubated with rRPTP/Phosphacan fused to hFc for 1 hr before fixation. Staining with anti‐Fc revealed colocalization of rRPTP/Phosphacan with Na_v_ clusters (Figure S5b). In addition, TNR and CNTN were also detected at prenodes in purified neuron cultures treated with OCM (Figure S5c,d).

We also tested the same multi‐molecular complexes or pre‐clustered rCNTN at 14 DIV for clustering activity. We found that these protein complexes had significant Na_v_ clustering activity (Figure 3g). In contrast to supplementing pre‐clustered rCNTN at 14 DIV, addition at 3 DIV to hippocampal neurons was not able to induce Na_v_ clustering due to possible toxic effect on neurons (not shown). Overall these results indicate that a multimolecular complex formed by CNTN with TNR and/or RPTP/Phosphacan can promote nodal protein clustering along hippocampal GABAergic axons.

### 3.4 Role of oligodendroglial‐secreted Contactin in Na_v_ cluster formation

**Figure 4:**
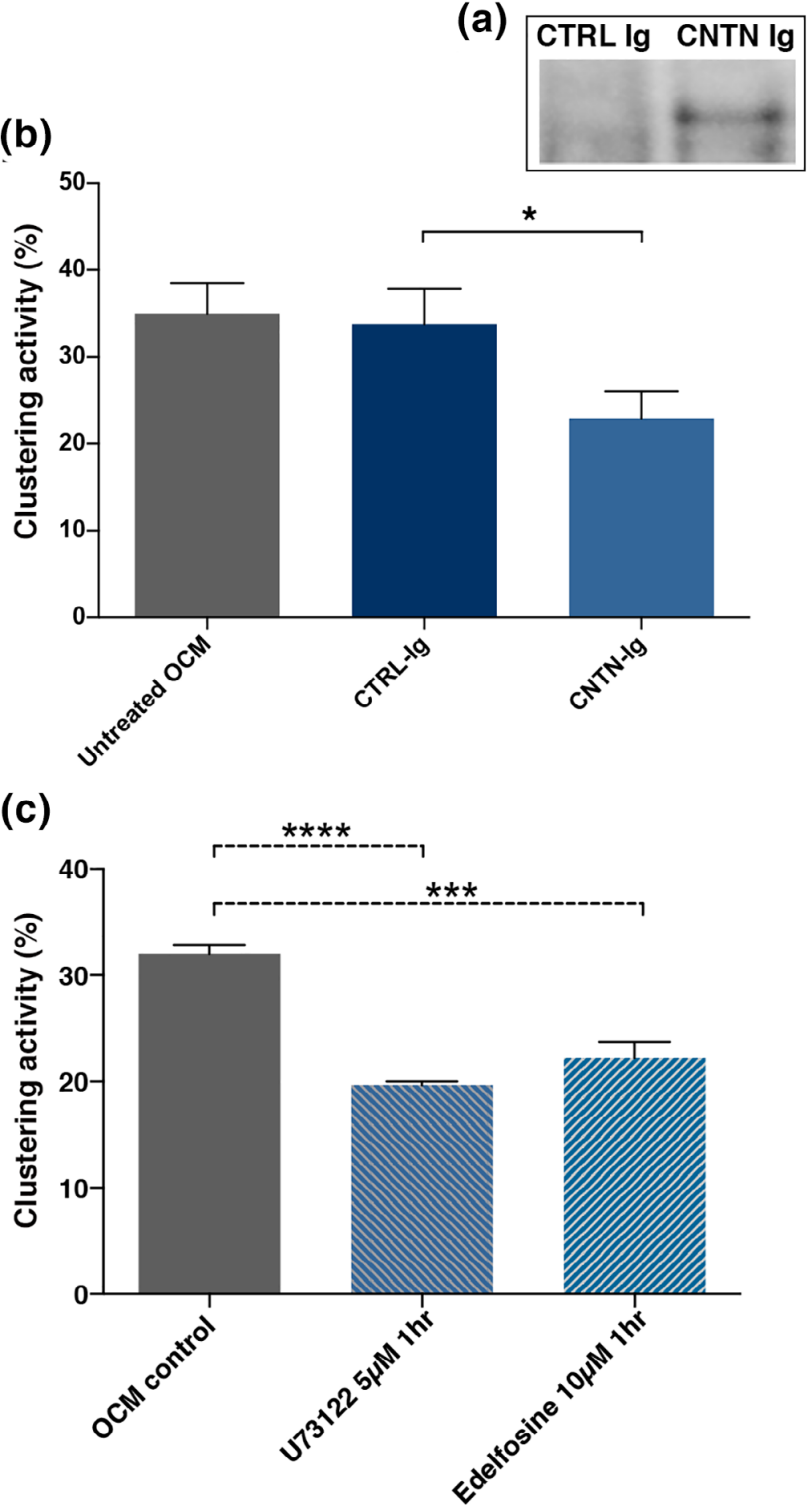
Depletion of CNTN from OCM by immunoprecipitation or phospholipase C inhibitors reduces its clustering activity. (a) CNTN immunoprecipitated from OCM using either anti CNTN‐IgG4 or CTRL‐IgG4 as shown by Western blot analysis with identification of CNTN in the precipitate; (b) Clustering activity of these OCMs added at 3 DIV to purified hippocampal neurons measured at 17 DIV, mean ± *SEM* of three different immunoprecipitated OCMs. Significant decrease of the clustering activity of anti‐CNTN treated OCM compared to both untreated OCM and CTRL treated OCM, *p = 0.02, paired *t*‐test. (c) OCM obtained from oligodendroglial cell cultures treated with phospholipase C inhibitors (U73122, 5 μM or Edelfosine, 10 μM) have a decrease clustering activity compared with control OCM; mean ± *SEM*, *n* = 3 and *n* = 4 different experiments, respectively; ****p < 0.0001; ***p = 0.0002, Dunn's multiple comparison test. OCM, oligodendrocyte conditioned medium; DIV, days in vitro; CNTN, contactin

To further probe the role of CNTN in OCM‐induced clustering, we used different strategies of CNTN depletion. We first performed immuno‐depletion experiments with an anti‐human CNTN Ab (Figure 4a) and compared the clustering activity of CNTN depleted OCM to control (i.e., non‐relevant) Ig‐treated OCM (Figure 4b). As shown in Figure 4b, a decrease of the clustering activity of anti‐CNTN treated OCM compared to both untreated OCM and CTRL‐Ig treated OCM was observed (mean ± *SEM*: 22.9 ± 3.1% vs. 35.0 ± 3.5%, and 33.8 ± 4.1%, respectively, *p* = 0.02). Of note, two successive immuno‐precipitations (IP) resulted in a similar reduction of clustering activity (~30%, not shown). Then, as CNTN is a GPI‐anchored glycoprotein which exists in membrane bound and soluble forms (Brümmendorf et al., 1993), and is susceptible to phosphatidylinositol‐phospholipase C (PI‐PLC) cleavage (Koch, Brugger, Bach, Gennarini, & Trotter, 1997), we inhibited its cleavage by adding PI‐PLC inhibitors (edelfosine or U73122) before harvesting OCM from oligodendrocyte cultures. As shown in Figure 4c, both PLC inhibitors, edelfosine or U73122, induced a *~* 30% reduction of Na_v_ clustering activity compared to OCM from untreated oligodendrocytes (mean ± *SEM*: 22.3 ± 1.5% and 19.7 ± 0.3% vs. 32% ± 0.9, *p* = 0.0002 and *p* < 0.0001, respectively).

**Figure 5:**
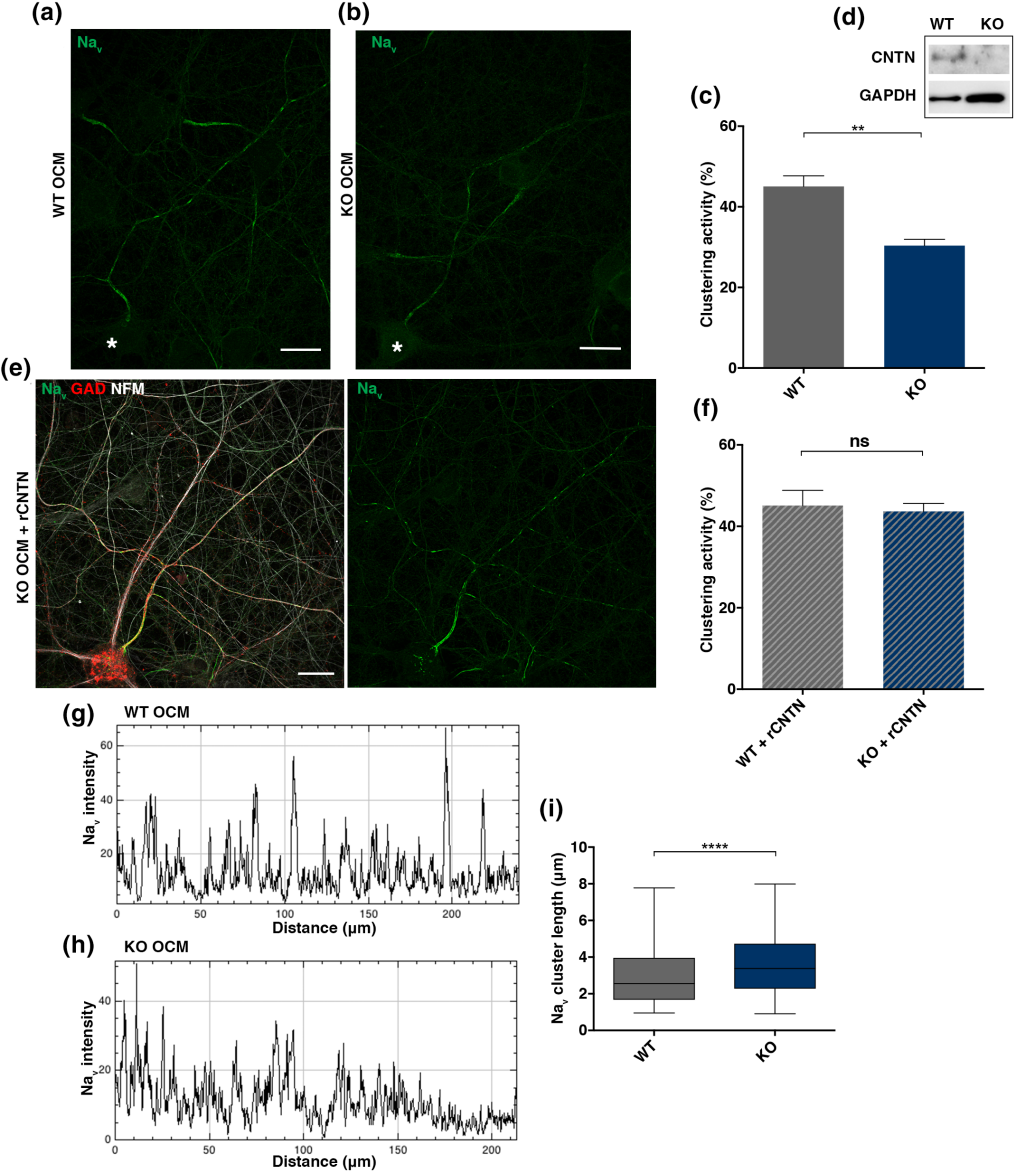
Genetic depletion of CNTN from OCM impacts clustering activity and shape of Na_v_ clusters. (a, b) Na_v_ immunostaining at 17 DIV (green) reveals clusters in neurons incubated either with OCM from *CNTN* ^*+/+*^ (WT‐OCM) or from *CNTN* ^*−/−*^ mice (KO‐OCM). *GABAergic neuron cell body. (c) Quantification of KO‐OCM clustering activity compared to WT‐OCM; mean ± *SEM* of three different KO‐ and WT‐ OCMs added on three different purified hippocampal neuron cultures, ** *p* = 0.0022, Mann Whitney test. (D) Western blot of CNTN from lysate of glial cell cultures from WT or KO animal (upper band), GAPDH as a load marker, confirming the absence of CNTN in the KO condition. (e) Immunostaining at 17 DIV showing well‐defined Na_v_ (green) clusters along a GABAergic axon (GAD67^+^, red) in culture treated with KO‐OCM and rCNTN at 3 DIV. (f) Addition of rCNTN in the medium restored Na_v_ clustering activity of KO‐OCM to levels indiscernible from WT‐OCM, Mann Whitney test, mean ± *SEM* of three different KO‐ and WT OCM added on three different purified hippocampal neuron cultures. (g, h) fluorescence intensity profiles corresponding to axonal Na_v_ immunolabeling from (a and b), Na_v_ clusters in KO‐OCM treated neurons are less restricted compared to WT‐OCM treated neurons. (i) Cluster lengths, measured at mid peak on Na_v_ plot profiles; >600 clusters measured in each condition in three different experiments, *p* < 0.0001, student *t*‐test. OCM, oligodendrocyte conditioned medium

To further address the specific role of oligodendroglial CNTN, OCM was produced from CNTN‐deficient oligodendrocytes isolated from the knockout mice (KO‐OCM) and clustering activity was compared to OCM from WT oligodendrocytes (WT‐OCM) in cultures of purified rat hippocampal neurons. We verified that CNTN was expressed in WT but not in KO glial cell culture lysate (Figure 5d). Na_v_ cluster formation was induced by WT‐OCM and KO‐OCM (Figure 5a,b), but a significant decrease (32.6%) of clustering activity was detected in KO‐OCM compared to WT‐OCM, (i.e., mean ± *SEM*: 30.4 ± 1.6% vs. 45.1 ± 2.6%, *p* = 0.002, respectively) (Figure 5c). To validate that the decreased clustering activity was due to the loss of CNTN, rCNTN was added to CNTN KO‐OCM, which restored the clustering activity, that is, KO‐OCM versus KO‐OCM + rCNTN (mean ± *SEM*: 30.4 ± 1.6% vs. 43.7 ± 1.9%, *p* = 0.0095, respectively) and WT‐OCM + rCNTN versus KO‐OCM + rCNTN (mean ± *SEM*: 45.1 ± 3.7 vs. 43.7 ± 1.9%, ns, respectively) (Figure 5e,f). In contrast, supplementing WT‐OCM with rCNTN did not further enhance clustering activity.

In addition, analysis of the Na_v_ profile along axons revealed that Na_v_ clusters were significantly longer on axons treated with CNTN KO‐OCM versus WT OCM (i.e., mean ± *SEM*: 3.65 μm ± 0.07 vs. 3.00 μm ± 0.06, respectively, *p* < 0.0001), suggesting an effect of CNTN on the spatial restriction of clusters (Figure 5g,h,i).

Altogether, these data indicate that oligodendroglial‐secreted contactin‐1 is critical in inducing CNS nodal protein clustering.

### 3.5 Role of Contactin in early Na_v_ clustering in vivo

**Figure 6:**
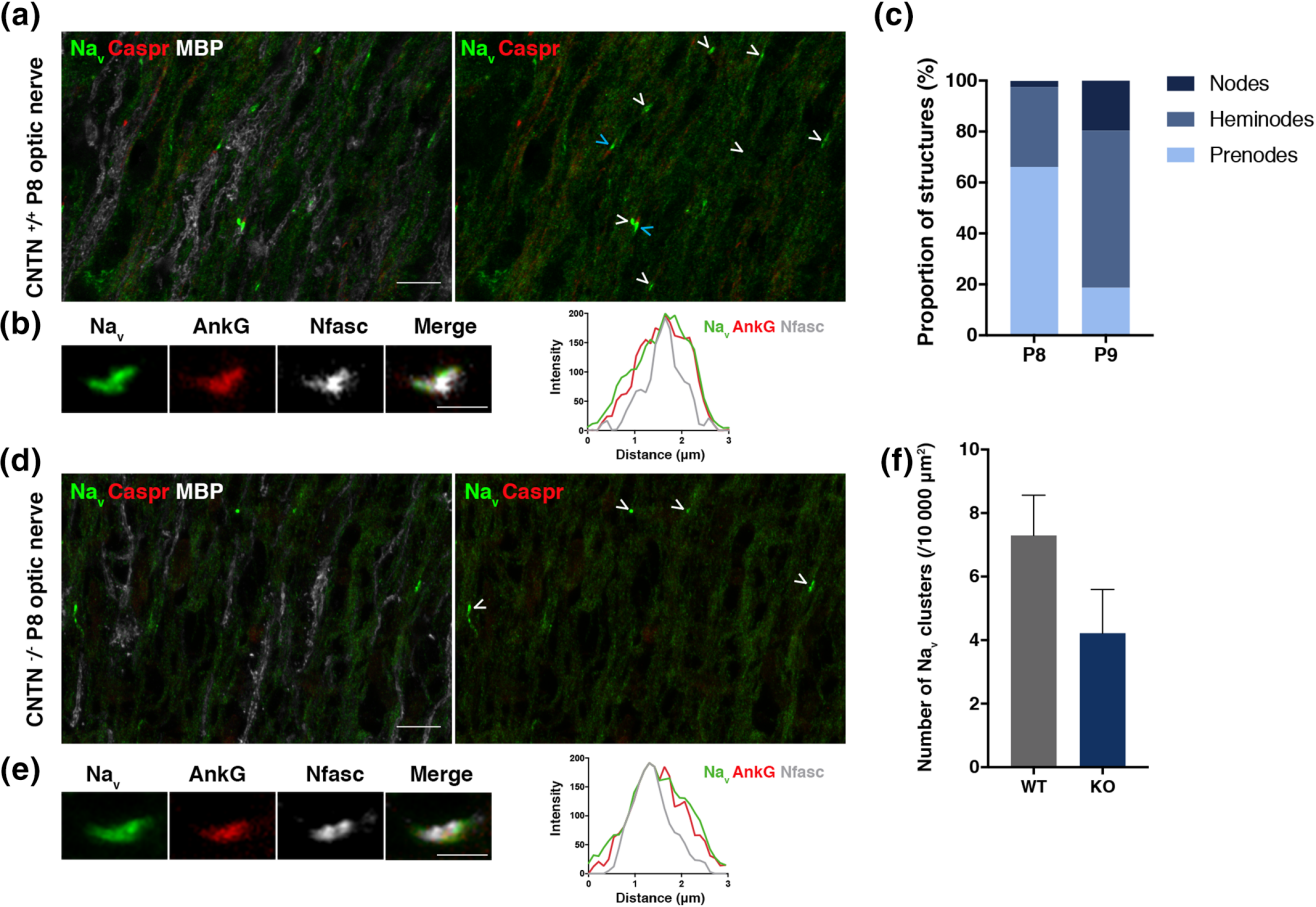
Impact of CNTN on early Na_v_ channel cluster formation in the optic nerve at P8. (a and d) immunostainings of sections of WT (a) and CNTN‐KO (D) P8 mice optic nerve show the presence of Na_v_ clusters (green), Caspr clusters (red) in the WT (a) but not the KO condition (d), and myelin (MBP^+^, white). Both heminodes (blue arrowhead) and prenodal clusters (white arrowhead) are observed in the WT (a). Scale bars: 10 μm. (b and r) immunostainings of sections of WT (B) and KO (E) P8 mice optic nerve show clusters with Na_v_ (green), AnkyrinG (red) and Nfasc (white) that are colocalizing (plot profile). Scale bars: 2 μm. (c) Quantification of the proportion of the different structures (i.e., nodes, heminodes, and prenodes or isolated Na_v_ clusters) from images of Na_v_ and Caspr immunostainings on WT optic nerve at P8 (*n* = 6 mice, 87 images) and P9 (*n* = 4 mice, 40 images). (f) Quantification of density of Na_v_ clusters per 10,000 μm^2^ for the WT and the KO at P8, mean ± *SEM*, student *t*‐test, ns. Values were obtained from *n* = 6 WT and *n* = 5 KO animals, with 10 to 20 images acquired per animal

We then addressed CNTN's contribution to nodal assembly in vivo in mice with inactivated gene expression (*CNTN* ^−/−^) (Berglund et al., 1999). As these mice display developmental abnormalities in the hippocampal formation (unpublished results from B. Ranscht) analysis of CNTN's impact on nodal assembly was investigated elsewhere. OCM‐induced Na_v_ clustering has been previously shown on retinal ganglion cells in vitro (M. R. Kaplan et al., 1997), and optic nerves present an ideal structure for Na_v_ cluster examination as no overt defects in innervating appropriate target regions were detected in the knockout condition (Çolakoğlu, Bergstrom‐Tyrberg, Berglund, & Ranscht, 2014). In WT mice, at P7 almost no Na_v_ clusters were detected (not shown), while they were distinct at P8 (Figure 6a). Notably, at P8 Na_v_ clusters were mainly detected in two states, in isolation or flanked on one side by paranodal Caspr (i.e., heminodal structure) (Figure 6a), and represented 66.1 ± 2.9% and 31.4 ± 2.4% of total Na_v_ clusters, respectively (Figure 6c; mean ± *SEM*, *n* = 6 mice, 87 images). In addition, at P8 other molecules of the nodal complex (i.e., AnkyrinG and Nfasc186) were also recruited and colocalized with Na_v_ in isolated clusters, as shown by plot profile tracing on acquired images (Figure 6b). The number of isolated clusters was decreased at P9 and represented 18.6 ± 1.7% of total Na_v_ clusters, while heminodes represented 61.7 ± 3.1% and nodes of Ranvier flanked on both side by paranodal Caspr 19.6 ± 3.5% (Figure 6c; mean ± *SEM*, *n* = 4 mice, 40 images).

In the KO condition, no Caspr clusters were detected (Figure 6d) as expected (Çolakoğlu et al., 2014). Na_v_ clusters were still formed and colocalized with AnkG and Nfasc (Figure 6c). We quantified the density of Na_v_ clusters at P8 and observed a 42.5% reduction of Na_v_ cluster density in the optic nerves of KO compared to the WT condition (i.e., number of Na_v_ clusters / 10,000 μm^2^, mean ± *SEM* from 6 WT and 5 KO animals; 7.3 ± 1.3 for the WT vs. 4.2 ± 1.4 for the KO condition, ns) (Figure 6f). Heterogeneity in Na_v_ clusters density among groups of WT as well as KO individuals may explain why this decrease is not statistically significant.

### 3.6 Role of neuronal NrCAM in restriction of Na_v_ clusters along axons

**Figure 7:**
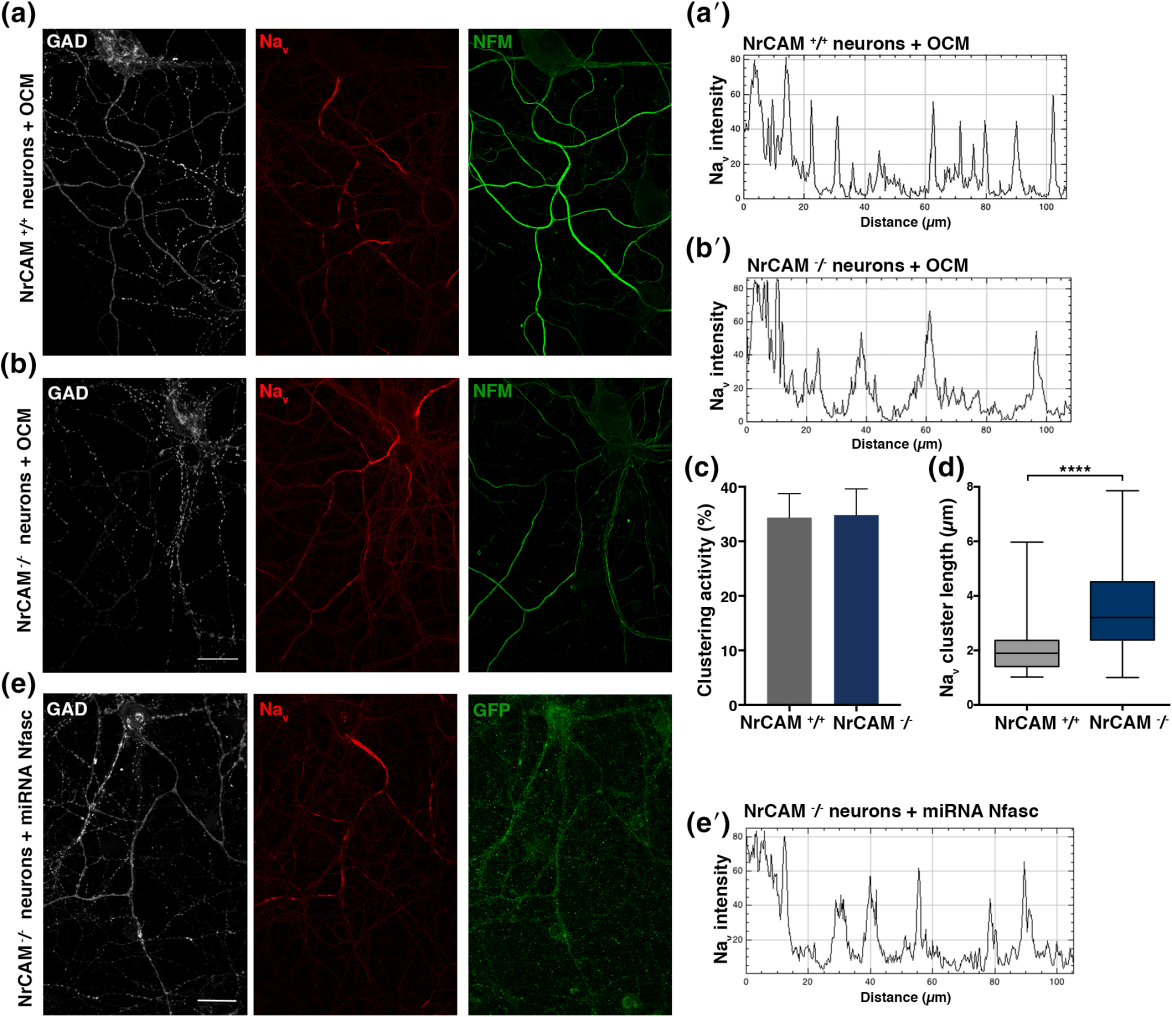
Formation of Na_v_ clusters in the absence of NrCAM and Nfasc186 Immunostainings of hippocampal purified neurons from *NrCAM* ^*+/+*^ (a) or *NrCAM* ^*−/−*^ (b) mice at 17 DIV, in cultures treated with WT OCM, showing clusters of Na_v_ (red) along a GAD67^+^ axon (white) expressing neurofilament M (NFM; green). Scale bars: 25 μm. Fluorescence intensity profiles (a′) and (b′) corresponding to Na_v_ immunolabeling from (a) and (b). Na_v_ clusters in NrCAM KO neurons are less restricted compared to NrCAM WT neurons. (c) Clustering activity of OCM added on WT or *NrCAM* KO purified hippocampal neurons measured at 17 DIV; mean ± *SEM* of 5 KO and 3 WT neuron cultures. (d) Cluster length, measured at mid peak on Na_v_ plot profiles; >600 clusters measured in each condition in three different experiments, *p* < 0.0001, Student *t*‐test. (e) Immunostaining of hippocampal cell cultures transfected at 6 DIV with Nfasc miRNA and fixed at 17 DIV. Representative image of a transfected neuron expressing GFP (green) and GAD67^+^ (white); Na_v_ expression (red) is detected at the AIS and clusters are observed. Scale bar: 25 μm (e′) Fluorescence intensity profiles corresponding to Na_v_ immunolabeling from (e). OCM, oligodendrocyte conditioned medium; DIV, days in vitro

To further characterize mechanisms of nodal protein assembly induced by CNTN, we determined the role of its neuronal partners Nfasc186 and NrCAM (Morales et al., 1993; Volkmer et al., 1998). We have previously shown that clusters are still formed on neurons from Nfasc186 deficient mice (*Nfasc186* ^−/−^) (Freeman et al., 2015). To investigate the role of NrCAM, we used purified hippocampal neuron cultures from *NrCAM* ^*−/−*^ mice. As illustrated in Figure 7b, Na_v_ clusters are formed along *NrCAM* ^*−/−*^ neurons treated with OCM, as well as with rCNTN and rRPTP/Phosphacan (Figure S6a–c). The OCM‐clustering activity was not significantly different in purified neuron cultures from WT versus *NrCAM*‐KO mice (mean ± *SEM* from three different WT and five different KO cultures, 34.3 ± 4.4% and 34.8 ± 4.8%, respectively) (Figure 7c), indicating that OCM activity was not impaired in the absence of neuronal NrCAM. However, as illustrated in Figure 7a,b and quantified on plot profiles (Figure 7a′,b′), clusters were significantly elongated in the absence of neuronal NrCAM (Figure 7d).

We then asked whether a functional compensation could exist between neuronal NrCAM and Nfasc186. To address this question, a knockdown of Nfasc186 was performed by specific miRNA expression in *NrCAM* ^−/−^ neurons. Transfected cells, identified by GFP expression, showed a strong reduction of Nfasc expression (Figure S6d). As illustrated in Figure 7e, Nfasc knockdown in *NrCAM* ^−/−^ neurons did not prevent Na_v_ clustering. However, the clusters formed were elongated (Figure 7e′). No differences were observed compared to the control condition, that is, *NrCAM* ^−/−^ neurons transfected with control miRNA (not shown).

In combination, these results indicate that neither NrCAM nor Nfasc186 are needed for nodal protein clustering. However, a role of neuronal NrCAM in the stabilization of the nodal protein complex is likely, as suggested by the lack of spatial restriction of nodal clusters in the absence of NrCAM.

## 4 DISCUSSION

We have identified oligodendrocyte‐secreted CNTN as a factor participating in the clustering of nodal proteins along axons. We provide evidence that a multi‐molecular complex consisting of CNTN combined with extra‐cellular matrix molecules RPTP/Phosphacan or TNR, promotes Na_v_ clustering on hippocampal neurons. These results are corroborated by the reduction of OCM‐induced Na_v_ clustering activity by CNTN immuno‐depletion and use of OCM from *Cntn1*‐deficient oligodendrocytes, and further validated by the demonstration that addition of recombinant CNTN to CNTN‐deficient OCM restores its full activity.

### 4.1 Mechanisms of nodal protein clustering induced by oligodendroglial Contactin

One of the first studies addressing Na_v_ clustering mechanism in the CNS concluded that oligodendroglial‐secreted soluble factors promote Na_v_ clustering on retinal ganglion cell axons (Kaplan et al., 1997). Similarly, our previous data provided evidence that on hippocampal GABAergic neurons, nodal assembly occurs under the influence of secreted cues, without the need of direct oligodendroglial contact (Freeman et al., 2015).

The present study provides evidence that soluble CNTN in combination with ECM proteins RPTP/Phosphacan or TNR triggers assembly of nodal complexes, consisting of Na_v_ channels, AnkyrinG, Nfasc186, and NrCAM. TNR and RPTP/Phosphacan have been previously shown to functionally interact with neuronal or glial CNTN isoforms (Brümmendorf et al., 1993; Peles et al., 1995; Rathjen et al., 1991; Zacharias et al., 1999). Such combinations could promote CNTN oligomerization, which would in turn increase the avidity of low‐affinity interactions for axonal receptors and induce Na_v_ clustering. Indeed, TNR forms trimeric structures that can function as molecular cross‐linkers to assemble the ECM (Lundell et al., 2004; Zacharias et al., 1999).

In the PNS, it has been shown that glial NrCAM modifies Gliomedin binding affinity to axonal Nfasc186 and Gliomedin‐NrCAM interactions with Nfasc186 are required to promote Nfasc186 and Na_v_ clustering at heminodes (Feinberg et al., 2010). In parallel, CNTN and NrCAM are present in OCM active fractions, however, their interactions are not required for developmental Na_v_ clustering as combinations of rCNTN and rNrCAM did not induce Na_v_ clustering on hippocampal axons.

Our results show that soluble CNTN is associated with proteins of the nodal complex. Neuronal Nfasc186 and NrCAM are expressed at nodes and known to be CNTN receptors (Morales et al., 1993; Volkmer et al., 1998). However, by miRNA mediated‐knock‐down of Nfasc186 in *NrCAM* ^−/−^ hippocampal neurons, we demonstrate that neither Nfasc186 (Freeman et al., 2015) nor neuronal NrCAM are critical for Na_v_ clustering. Nevertheless, as clusters are significantly elongated in the absence of oligodendroglial CNTN or neuronal NrCAM, CNTN interactions with NrCAM may have a role in stabilizing prenodes.

CNTN binding to other neuronal CAMs may promote prenodal clustering activity. Although CNTN does not engage in direct homophilic binding (Faivre‐Sarrailh, Falk, Pollerberg, Schachner, & Rougon, 1999), interactions with RPTP/Phosphacan or TNR may bridge neuronal and OCM‐derived CNTN. In addition, ß1 and ß2 Na_v_ subunits, which are structurally very close to the family of CAMs (Catterall, 2010; Chopra, Watanabe, Zhong, & Roden, 2007; Srinivasan, Schachner, & Catterall, 1998), have been reported to interact in *cis* or *trans* with CNTN, TNR or RPTP/phosphacan (Srinivasan et al., 1998; Xiao et al., 1999). This interaction results in increased Na_v_1.2 cell surface density and modulation of Na_v_1.2 current (Kazarinova‐Noyes et al., 2001; McEwen, Meadows, Chen, Thyagarajan, & Isom, 2004; Xiao et al., 1999). Therefore, Na_v_ß subunits may represent other axonal partners mediating nodal protein clustering, as suggested by a recent study from our laboratory (Thetiot et al., submitted).

Finally, loss of AnkyrinG results in significant alterations in CNS nodal and prenodal clustering in hippocampal neurons (Freeman et al., 2015; Jenkins et al., 2015; Susuki et al., 2013). CNTN and RPTP/Phosphacan or TNR binding to CAMs or Na_v_ ß subunits could serve to recruit AnkyrinG with subsequent anchoring of the complex to the underlying cytoskeleton through interactions with ßIV spectrin (Lustig et al., 2001; Malhotra et al., 2002).

### 4.2 Neuronal maturation occurs prior to myelination but is not sufficient to induce Na_v_ clustering

Our results suggest that OCM induces axonal growth and maturation of hippocampal neurons, as indicated by increased neurofilament M expression (Shaw & Weber, 1982). This emphasizes the essential role of oligodendrocytes in providing complex trophic signals and/or metabolic support to neurons (Meyer‐Franke, Kaplan, Pfrieger, & Barres, 1995; Sánchez, Hassinger, Paskevich, Shine, & Nixon, 1996; Wilkins, Chandran, & Compston, 2001; Wilkins, Majed, Layfield, Compston, & Chandran, 2003). The study by Kaplan et al. (2001) also demonstrates that axonal maturation is associated to early nodal protein clustering on retinal ganglion cells prior to myelination. This is consistent with our results which indicate that hippocampal neurons respond with cluster formation to OCM addition with a delay, suggesting that neuronal maturation is necessary for the Na_v_ clustering process. This supports the hypothesis that OCM may induce complex intracellular signaling rather than simple mechanic clustering of proteins at the axon surface. Although neuronal maturation is a prerequisite for Na_v_ clustering, it is not sufficient to induce Na_v_ clustering as indicated by the lack of prenodes on neurons induced to mature by the addition of growth factors. Our results are in line with early cytochemical and freeze fracture studies which have provided evidence for the differentiation of the axon prior to formation of compact myelin in spinal nerve roots (Waxman & Foster, 1980) and demonstrated for the first time, in rat developing optic nerve axons, clusters of intramembranous particles on the external face in some fibers prior to the formation of any compact myelin (Waxman, Black, & Foster, 1982).

### 4.3 Na_v_ cluster assembly in vivo

In the mouse optic nerve, oligodendrocytes development and myelination begin at the start of the second postnatal week (Black, Foster, & Waxman, 1982; Skoff, Price, & Stocks, 1976). Our data in the WT mouse optic nerve indicate that as early as P8, isolated Na_v_ clusters (i.e., without flanking Caspr immunoreactivity) are mostly detected. They colocalize with AnkyrinG and Nfasc186, forming prenodes or nodal‐like clusters prior to paranodal junction assembly. We show that these prenodes are only transiently present during development (their proportion being strongly decreased at P9), suggesting a very dynamic maturation of the optic nerve in the second postnatal week. Our data are consistent with previous studies in the rat optic nerve, showing Na_v_ clusters at P9‐P10 (Rasband et al., 1999), including a small proportion of isolated structures without flanking Caspr immunoreactivity (Rasband et al., 1999). In addition to the optic nerve and the hippocampus (Freeman et al., 2015), prenodes have been detected in Purkinje cells in the cerebellum (Anne Desmazieres, unpublished results). What distinguishes axons that form prenodes might be their differentiation state and might be associated with the need for early establishment of neuronal connections during development of axons with long trajectories (Black et al., 1982; Freeman et al., 2015; Jinno et al., 2007). Physiological specialization linked to nodal structures has been previously shown along fibers in the neurogenic electric organ of the knife fish Sternarchus (Waxman, Pappas, & Bennett, 1972).

### 4.4 Relevance of Contactin for nodal assembly in vivo

In the *Cntn1‐* KO optic nerves, whereas paranodes are disrupted, as indicated by the absence of Caspr and as previously demonstrated (Boyle et al., 2001; Çolakoğlu et al., 2014), we show the presence of Na_v_ clusters. Our data are consistent with previous studies demonstrating that paranodal junction formation is not essential for initial Na_v_ clustering in the CNS, as reported in mutants with disrupted paranodes, that is, in Caspr‐, CGT‐ and CST‐KO mice (Bhat et al., 2001; Ishibashi et al., 2002; Rios et al., 2003; Susuki et al., 2013). Our data indicating a decrease of about 40% of mean density of Na_v_ clusters in *Cntn1*‐KO optic nerve compared to WT suggest that oligodendroglial CNTN may participate to nodal protein clustering in vivo. In addition to inter‐individual heterogeneity of Na_v_ clusters, potential redundancy with other ECM molecules (i.e., Brevican, Versican, Bral1) or clustering induced through AnkyrinG interactions may explain that this reduction of Na_v_ clustering is only partial.

High level of CNTN expression is detected in oligodendrocyte progenitor cells and differentiating oligodendrocytes (Çolakoğlu et al., 2014). CNTN binds both to RPTP/Phosphacan and TNR (Peles et al., 1995; Rathjen et al., 1991), suggesting possible formation of a multi‐molecular complex which participates in early nodal clustering in vivo. Electron microscopic analysis revealed TNR immunoreactivity at contact sites between axons and processes of oligodendrocytes at P13/P14 (Bartsch, Bartsch, Dörries, & Schachner, 1992). Moreover, it has been shown that perinodal astrocytes and oligodendrocyte precursor cells extend processes that contact some nodes, which may participate in the formation of mature central node and/or stabilization (Black & Waxman, 1988; Butt et al., 1999; Ffrench‐Constant, Miller, Kruse, Schachner, & Raff, 1986; Serwanski, Jukkola, & Nishiyama, 2017). In addition, in situ hybridization analysis of RPTP‐ß expression in the developing rat brain revealed high level of phosphacan transcript (i.e., 8.4‐kb transcript) in glial cells that have migrated out of the proliferative zone (Canoll, Petanceska, Schlessinger, & Musacchio, 1996). Notably, a strong signal was detected in the stratum oriens of the hippocampus at P7 (Canoll et al., 1996), a region where prenodes were observed (Freeman et al., 2015). Altogether, these in vivo observations support our hypothesis that an early dialogue between axons and oligodendrocyte precursors or differentiating oligodendrocytes is capable of eliciting Na_v_ clustering along axons. The results from this study are in line with the suggestion that CNTN is one of the contributing molecules in this early communication.

## AUTHOR CONTRIBUTIONS

Conceptualization, AL.D., C.L., and N.SF.; Methodology, AL.D., M.Fi., M.Fl., and N.SF.; Formal analysis, AL.D., E.M., S.O., and N.SF.; Investigation, AL.D., Q.R., E.M., S.O., M.T. and E.C.; Resources, MS.A, B.R., C.A.G.; Writing – Original Draft, AL.D., C.L., and N.SF.; Writing – Review & Editing, AL.D., E.M., A.D., R.K., B.Z., B.R., C.A.G., C.L., and N.SF., Supervision, R.K., B.Z., C.L. and N.SF.

## Supporting information


**Figure S1** OCM as well as growth factors (GF) ‐i.e., IGF‐1, BDNF and GDNF‐ added at 3 DIV on purified hippocampal neuron cultures increase axonal length (measured at 6 DIV) of both GABA and non‐GABAergic neurons, compared to control medium (CTRL). Values are the mean length in μm ± SEM of 15 to 30 axons per condition in 3 different experiments, (p < 0.0001 when comparing conditions calculated with ANOVA test, multiple comparisons performed with Dunn's multiple comparisons test).

**Figure S2** Oligodendroglial maturation does not influence OCM‐clustering activity. (A) Illustrative image of oligodendroglial cultures in standard condition showing the coexistence of OPCs (NG2^+^ cells; red), immature oligodendrocytes (NG2^+^ and O4^+^ cells; red and green) and premyelinating oligodendrocytes (O4^+^ cells; green, with bushy morphology). **(B)** Quantification of cell phenotype in cultures depending on positivity of NG2, O4, and PLP. Compared to standard condition, OPC‐enriched cultures (treated with 100 nM rapamycin) have increased number of NG2^+^ cells, that is, 71% for standard versus 82% for OPC‐enriched cultures, and decreased number of O4+ cells, that is, 50.2 versus 32%, respectively, (n = 3 different cultures); mean ± SEM. Cultures obtained from PLP‐GFP animals after FACS‐sorting contained 90% of PLP^+^ oligodendrocytes versus 3.8% in standard condition and no immature OPC, (*n* = 2 FACS‐sorting experiments). **(C)** Clustering activity of different conditioned media added to purified hippocampal neurons, normalized to OCM obtained in standard condition. Standard OCM, 100%, OPC enriched–CM, 88%, and mature OL‐CM, 92%, which represent the mean ± SEM of three different OPC‐enriched‐CM and two different mature OL‐CM added on two different purified hippocampal neuron cultures.

**Figure S3 Clustering activity of control medium and OCM fractions. (A)** Clustering activity of non‐fractionated (NF) and fractionated OCM (A9 to B13, blue bars) or control medium not incubated with oligodendroglial cells (A9 to B13, red bars) measured on purified hippocampal neurons at 17 DIV. Active OCM fractions (green rectangle) and inactive fractions (blue rectangle) used for proteomic analysis.

**Figure S4 Protein list obtained from the proteomic analysis of OCM fractions. (A)** Proteins identified in the active fraction F13 **(B)** Proteins identified in the inactive fraction F15. Indicated parameters are the percent coverage of the total protein sequence by the identified peptides (∑ coverage), the number of identified peptides that are unique to this protein (no overlap with other proteins in the database) (∑# Unique Peptides) and the Peptide Spectral Match (∑# PSMs), i.e., indicating how many times the mass spectra match that of the peptide sequence.

**Figure S5 Prenodes are induced by addition of rCNTN and rTNR, and phosphacan or TNR are stabilized at prenodes on OCM treated neurons. (A)** Immunostaining of neurons treated with rCNTN and rTNR at 3 DIV showing Na_v_ clusters (red) along GABAergic axon (GAD67^+^; green). **(B)** Incubation of OCM‐treated neuronal cultures with rRPTP/phosphacan for 1 h before fixation at 17 DIV. Immunostaining with an anti‐human Fc (green) reveals the binding of rRPTP/phosphacan and indicates the existence of binding partners at Na_v_ clusters. **(C)** Immunostaining of OCM‐treated hippocampal neuron culture with anti‐TNR (red) and anti‐AnkG (green). Scale bars: 25 μm. (**D**) Immunostaining of hippocampal neuron showing Na_v_ clusters (green), colocolazing with CNTN (human anti‐CNTN IgG4; red; upper part) in the absence of myelin anti‐PLP (white; negative). No signal was observed with a primary control human IgG4 (red; lower part).

**Figure S6 Addition of CNTN and RPTP/phosphacan on *NrCAM*^−/^^−^ purified neurons induces prenode formation. (A)** Na_v_ clusters are induced by rCNTN + rRPTP/phosphacan in the absence of NrCAM expression. Immunostainings of hippocampal purified neurons from *NrCAM* ^*−/−*^ mice at 17 DIV, in cultures treated with rCNTN + rRPTP/phosphacan, showing clusters of Na_v_ (green) and Nfasc (red) along a GAD67^+^ axon (white). Scale bars: 25 μm. **(B)** Fluorescence intensity profile corresponding to Na_v_ immunolabeling from (A). **(C)** Fluorescence intensity profile corresponding to Nfasc immunolabeling from (A). (D) Immunostainings for Na_v_ (white) and Nfasc (red) on hippocampal neuron culture transfected with miRNA Nfasc, showing a transfected neuron expressing GFP (green), and two neurons that were not transfected (GFP negative). White stars indicate the cell bodies; the framed region includes the transfected neuron. Na_v_ (white) is expressed at all AIS. In contrast, Nfasc is expressed at the AIS in GFP negative neurons (not transfected) but poorly detected in GFP positive neuron (expressing miRNA Nfasc).
