# Supplementary figures and images for: Role of a Contactin multi‐molecular complex secreted by oligodendrocytes in nodal protein clustering in the CNS
Source: Glia. 2019 Jul 22;67(12):2248–63. doi: 10.1002/glia.23681 (PMC6851800; doi:10.1002/glia.23681)

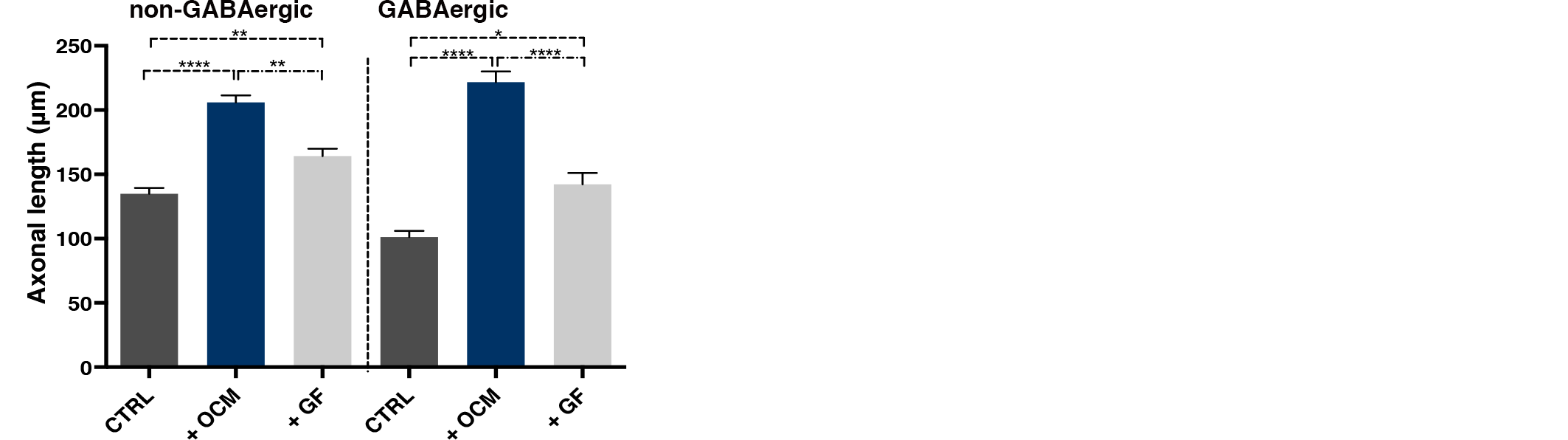

Supplement: Supplementary file 1 — Figure S1 OCM as well as growth factors (GF) ‐i.e., IGF‐1, BDNF and GDNF‐ added at 3 DIV on purified hippocampal neuron cultures increase axonal length (measured at 6 DIV) of both GABA and non‐GABAergic neurons, compared to control medium (CTRL). Values are the mean length in μm ± SEM of 15 to 30 axons per condition in 3 different experiments, (p < 0.0001 when comparing conditions calculated with ANOVA test, multiple comparisons performed with Dunn's multiple comparisons test). [file GLIA-67-2248-s001.tif]

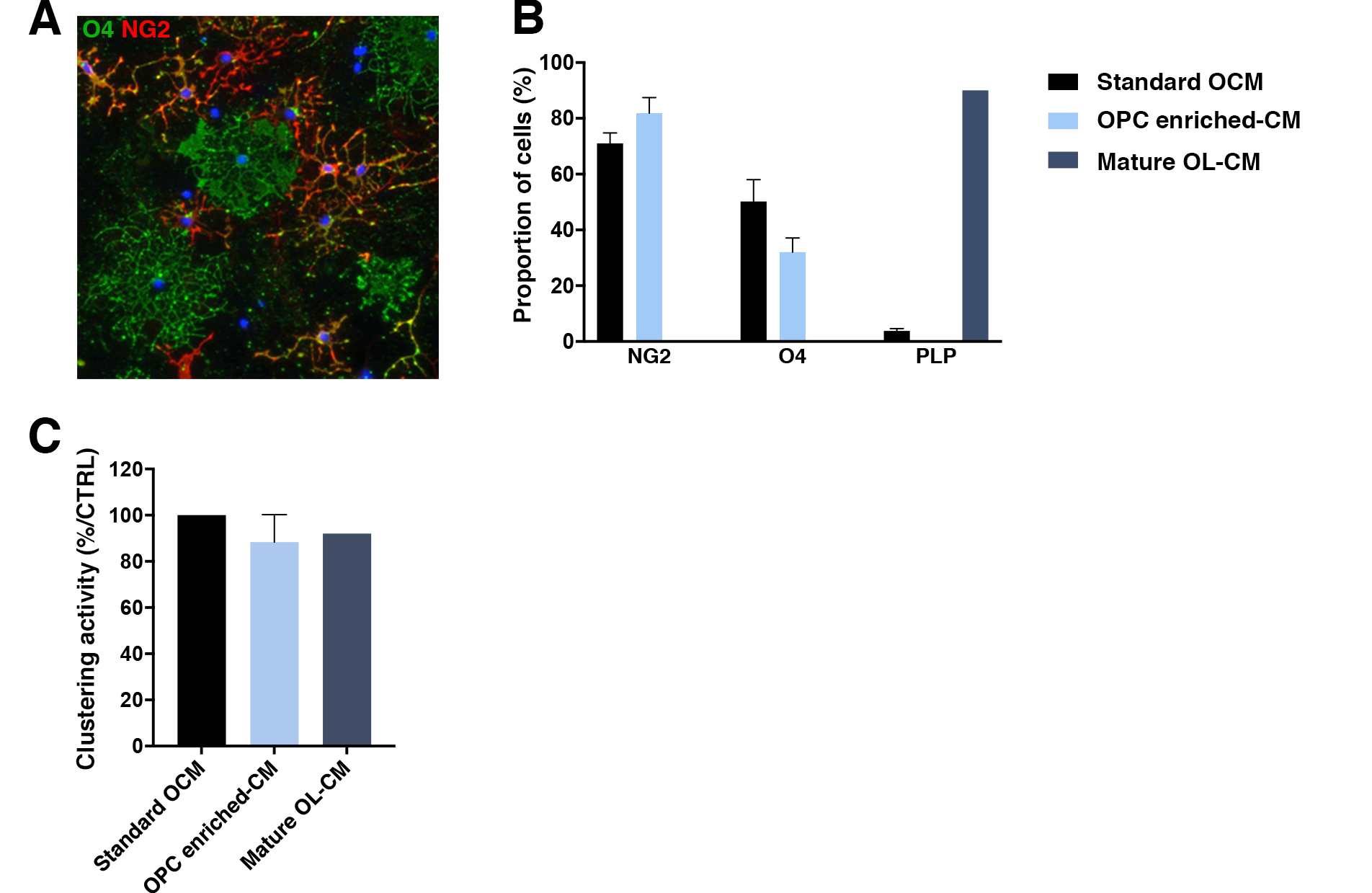

Supplement: Supplementary file 2 — Figure S2 Oligodendroglial maturation does not influence OCM‐clustering activity. (A) Illustrative image of oligodendroglial cultures in standard condition showing the coexistence of OPCs (NG2+ cells; red), immature oligodendrocytes (NG2+ and O4+ cells; red and green) and premyelinating oligodendrocytes (O4+ cells; green, with bushy morphology). (B) Quantification of cell phenotype in cultures depending on positivity of NG2, O4, and PLP. Compared to standard condition, OPC‐enriched cultures (treated with 100 nM rapamycin) have increased number of NG2+ cells, that is, 71% for standard versus 82% for OPC‐enriched cultures, and decreased number of O4+ cells, that is, 50.2 versus 32%, respectively, (n = 3 different cultures); mean ± SEM. Cultures obtained from PLP‐GFP animals after FACS‐sorting contained 90% of PLP+ oligodendrocytes versus 3.8% in standard condition and no immature OPC, (n = 2 FACS‐sorting experiments). (C) Clustering activity of different conditioned media added to purified hippocampal neurons, normalized to OCM obtained in standard condition. Standard OCM, 100%, OPC enriched–CM, 88%, and mature OL‐CM, 92%, which represent the mean ± SEM of three different OPC‐enriched‐CM and two different mature OL‐CM added on two different purified hippocampal neuron cultures. [file GLIA-67-2248-s002.tif]

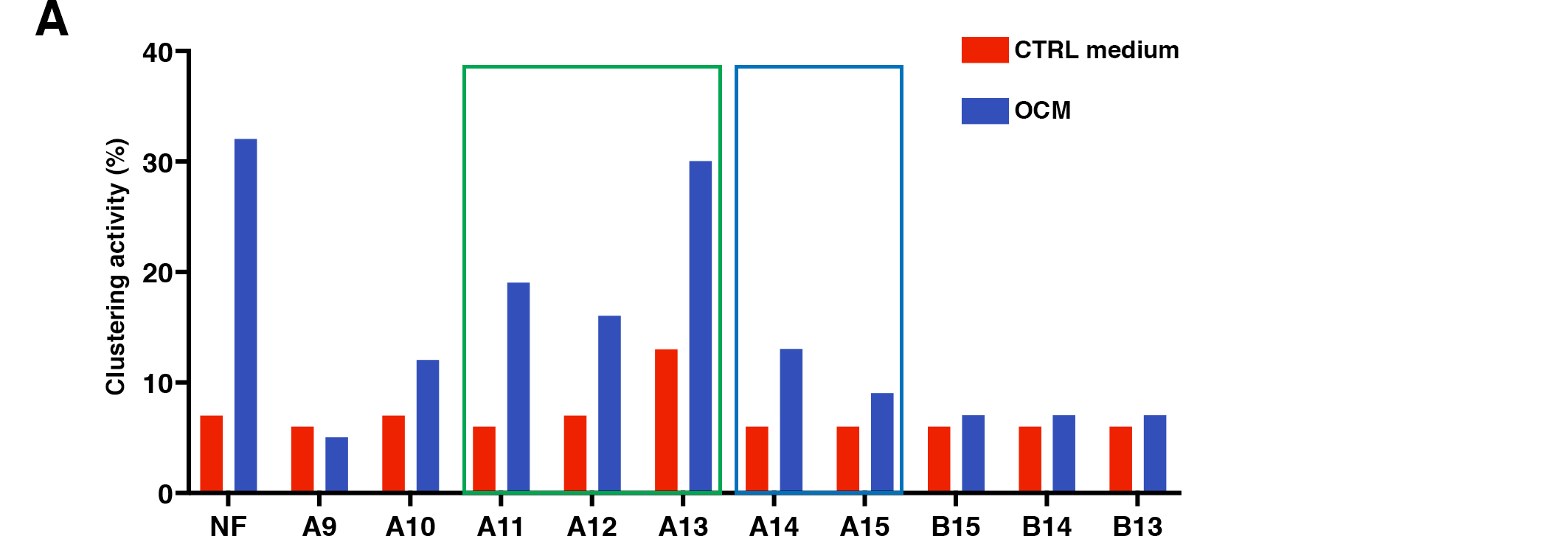

Supplement: Supplementary file 3 — Figure S3 Clustering activity of control medium and OCM fractions. (A) Clustering activity of non‐fractionated (NF) and fractionated OCM (A9 to B13, blue bars) or control medium not incubated with oligodendroglial cells (A9 to B13, red bars) measured on purified hippocampal neurons at 17 DIV. Active OCM fractions (green rectangle) and inactive fractions (blue rectangle) used for proteomic analysis. [file GLIA-67-2248-s003.tif]

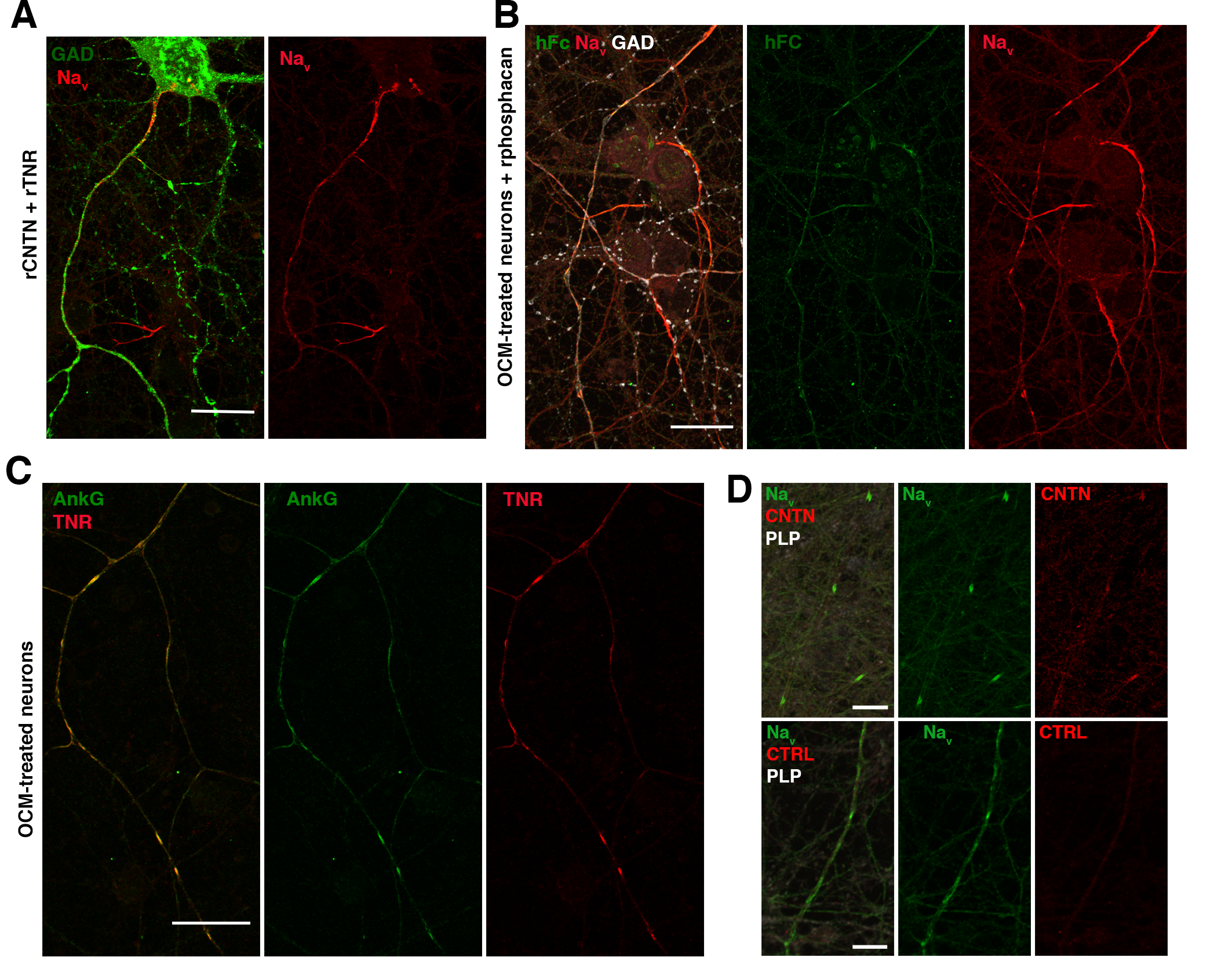

Supplement: Supplementary file 5 — Figure S5 Prenodes are induced by addition of rCNTN and rTNR, and phosphacan or TNR are stabilized at prenodes on OCM treated neurons. (A) Immunostaining of neurons treated with rCNTN and rTNR at 3 DIV showing Nav clusters (red) along GABAergic axon (GAD67+; green). (B) Incubation of OCM‐treated neuronal cultures with rRPTP/phosphacan for 1 h before fixation at 17 DIV. Immunostaining with an anti‐human Fc (green) reveals the binding of rRPTP/phosphacan and indicates the existence of binding partners at Nav clusters. (C) Immunostaining of OCM‐treated hippocampal neuron culture with anti‐TNR (red) and anti‐AnkG (green). Scale bars: 25 μm. (D) Immunostaining of hippocampal neuron showing Nav clusters (green), colocolazing with CNTN (human anti‐CNTN IgG4; red; upper part) in the absence of myelin anti‐PLP (white; negative). No signal was observed with a primary control human IgG4 (red; lower part). [file GLIA-67-2248-s005.tif]

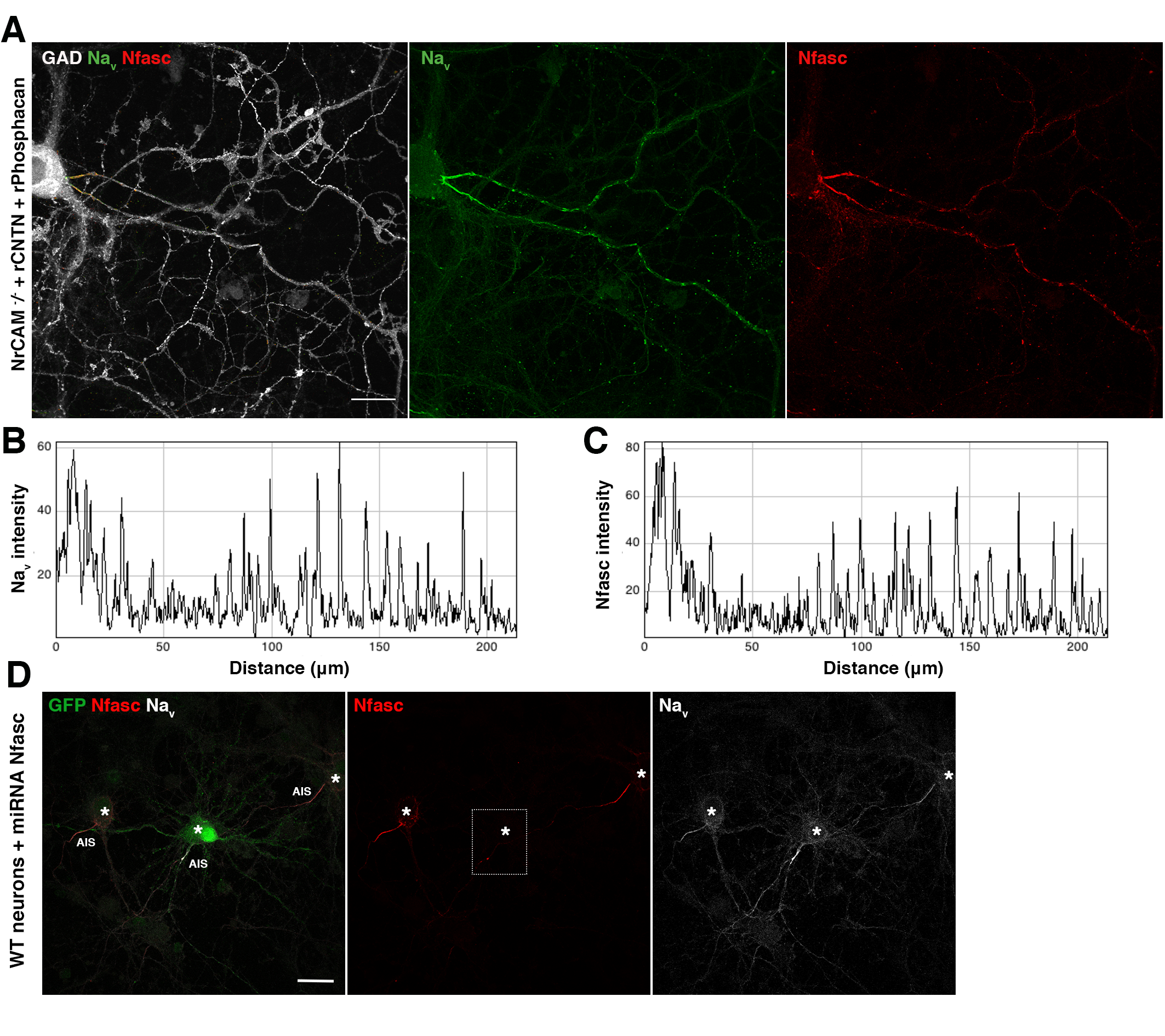

Supplement: Supplementary file 6 — Figure S6 Addition of CNTN and RPTP/phosphacan on NrCAM−/− purified neurons induces prenode formation. (A) Nav clusters are induced by rCNTN + rRPTP/phosphacan in the absence of NrCAM expression. Immunostainings of hippocampal purified neurons from NrCAM −/− mice at 17 DIV, in cultures treated with rCNTN + rRPTP/phosphacan, showing clusters of Nav (green) and Nfasc (red) along a GAD67+ axon (white). Scale bars: 25 μm. (B) Fluorescence intensity profile corresponding to Nav immunolabeling from (A). (C) Fluorescence intensity profile corresponding to Nfasc immunolabeling from (A). (D) Immunostainings for Nav (white) and Nfasc (red) on hippocampal neuron culture transfected with miRNA Nfasc, showing a transfected neuron expressing GFP (green), and two neurons that were not transfected (GFP negative). White stars indicate the cell bodies; the framed region includes the transfected neuron. Nav (white) is expressed at all AIS. In contrast, Nfasc is expressed at the AIS in GFP negative neurons (not transfected) but poorly detected in GFP positive neuron (expressing miRNA Nfasc). [file GLIA-67-2248-s006.tif]
