# Supplementary material for: Role of a Contactin multi‐molecular complex secreted by oligodendrocytes in nodal protein clustering in the CNS
Source: Glia. 2019 Jul 22;67(12):2248–63. doi: 10.1002/glia.23681 (PMC6851800; doi:10.1002/glia.23681)
Supplement: Supplementary file 4 — Figure S4 Protein list obtained from the proteomic analysis of OCM fractions. (A) Proteins identified in the active fraction F13 (B) Proteins identified in the inactive fraction F15. Indicated parameters are the percent coverage of the total protein sequence by the identified peptides (∑ coverage), the number of identified peptides that are unique to this protein (no overlap with other proteins in the database) (∑# Unique Peptides) and the Peptide Spectral Match (∑# PSMs), i.e., indicating how many times the mass spectra match that of the peptide sequence. [file GLIA-67-2248-s004.zip › glia23681-sup-0004-FigureS4A.docx]

List of identified proteins in F13 (active fraction)
